# Adults in Ghana generate higher and more durable neutralising antibody titres following primary course COVID-19 vaccination than matched UK adults: The HERITAGE Study

**DOI:** 10.1186/s12916-025-04157-0

**Published:** 2025-05-28

**Authors:** Eliza Mari Kwesi-Maliepaard, Yakubu Alhassan, Emmanuel K. Quaye, Vera M. Kotey, Aisha M. Mohammed, Seth Agyemang, Adelaide K. Sromani, Stephanie Darko, Erica Buadii, Randy Tackie, Harry Akligoh, Barikisu Ibrahim, David Hutchful, Lily Paemka, Emmanuella Amoako, Joyce M. Ngoi, Aida Manu, the HERITAGE study team, Emmanuel Agbeli, Wisdom Akotia, Susan Amoako, Apetsi Ampiah, Charles Ansong, Seyram B Atukpa, Wisdom Aveey, Frank Danquah, Stephen L Darkoh, Patricia Kaba, Ruth Kiome, Esmy Kotey, Silas Lawer, David Greenwood, Edward J. Carr, Mary Y. Wu, David L. V. Bauer, Emma C. Wall, the Crick Legacy Consortium, Lorin Adams, Karen Ambrose, Philip Bawumia, James Bazire, Rupert Beale, Bobbi Clayton, Tumena Corrah, Giulia Dowgier, Ashley S Fowler, Joshua Gahir, Steve Gamblin, Sonia Gandhi, Richard Gilson, Ruth Harvey, Agnieszka Hobbs, George Kassiotis, Gavin Kelly, Sven Kjar, Vincenzo Libri, Murad Miah, Mauro Miranda, Nicola O’Reilly, Padmasayee Papineni, Callie Smith, Phoebe Stevenson-Leggett, Charles Swanton, Scott Warchal, Bryan Williams, Dzifa Dey, Abdul Razak Quao, Akosua Ayisi, Kwame Amponsa-Achiano, Franklin Asiedu Bekoe, Gordon Awandare, Peter K. Quashie, Yaw Bediako

**Affiliations:** 1 Yemaachi Biotech, Accra, Ghana; 2 Department of Biostatistics, School of Public Health, University of Ghana, Accra, Ghana; 3 West African Centre for Cell Biology of Infectious Pathogens (WACCBIP), University of Ghana, Accra, Ghana; 4 The Francis Crick Institute, London, UK; 5 University College London, Gower St, London, UK; 6 National Institute for Health Research (NIHR) University College London Hospitals (UCLH) Biomedical Research Centre, London, UK; 7 NIHR UCLH Clinical Research Facility, London, UK; 8 University of Ghana Medical School, Korle Bu Teaching Hospital, Accra, Ghana; 9 Adabraka Polyclinic, Accra, Ghana; 10 Ghana Health Service, Accra, Ghana

**Keywords:** SARS-CoV-2, COVID-19 vaccine, Africa, Vaccine response, Geographical variation

## Abstract

**Background:**

Little data exist on the COVID-19 vaccine response in African countries who despite having high disease burden, have low COVID-19 mortality rates. We investigated the longitudinal immune response in a West-African urban population upon COVID-19 vaccination, two years after the start of the pandemic.

**Methods:**

The HERITAGE study is a prospective cohort study of 301 residents of Accra, Ghana. Participants received two doses of a COVID-19 vaccine (AZD1222 or BNT162b2) from December 2021 and were followed-up for 12 months. COVID-19 status was determined by RT-PCR at seven time points. Serological responses, including anti-Nucleocapsid IgG, anti-Spike IgG and live-virus neutralisation were determined at four time points during the 12 months follow-up.

**Results:**

COVID-19 positivity was 19.3% at baseline and reduced rapidly upon vaccination. Serological analyses indicated previous exposure to SARS-CoV-2 in 80.5% of the HERITAGE participants. After vaccination, neutralising antibody titres (NAbTs) against six different SARS-CoV-2 variants significantly (*p* < 0.001) increased, with fold changes (FC) ranging from 1.87 to 4.59. Highest NAbTs were recorded in the previously exposed group. Participants without prior exposure showed a continues increase in NAbTs between months 3 and 12 for circulating variants (Omicron B.A2 (FC 2.44, *p* < 0.001) and XBB.1.5 (FC 1.91, *p* = 0.05)). By comparison a matched cohort from the UK-based LEGACY study showed generally lower NAbTs at baseline (HERITAGE vs LEGACY for Wild-type: 250.3 vs 141.3, *p* < 0.0001, for A.27 84.6 vs 43.2, *p* = 0.0129, for Eta 159.7 vs 118.1, *p* = 0.3428, for Delta 158.6 vs 10.0, *p* < 0.0001, for Omicron B.A2 153.7 vs 10.0, *p* < 0.0001) and after receiving the vaccine (HERITAGE vs LEGACY for Wild-type: 882.6 vs 337.7, *p* < 0.0001, for A.27 552.0 vs 227.7, *p* = 0.0001, for Eta 682.2 vs 295.3, *p* < 0.0001, for Delta 557.6 vs 165.1, *p* < 0.0001, for Omicron B.A2 283.3 vs 124.2, *p* < 0.0001). NAbTs kinetics between the two cohorts were more similar when analysis was restricted to previously unexposed participants when adjusted for circulating variants during the sampling period.

**Conclusions:**

Two doses of AZD1222 or BNT162b2 significantly increased existing NAbTs against SARS-CoV-2 in a highly exposed population, showing durable boosting of pre-existing infection-induced immunity. This indicates the importance of considering local population exposure in vaccination design and deployment.

**Supplementary Information:**

The online version contains supplementary material available at 10.1186/s12916-025-04157-0.

## Background

The impact of the COVID-19 pandemic has differed between geographical areas, with overlapping as well as differential variant waves sweeping through different regions. Most research on COVID-19 takes place in high-income countries (HICs) in Europe and North America, where the majority of COVID-19 deaths are reported [1]. In low-and middle-income countries (LMICs), especially in Africa, the reported rates of both mortality and hospitalisation are lower despite limited availability and later deployment of COVID-19 vaccines [1, 2]. Several factors have been suggested to contribute to the lower mortality and hospitalisation rates in Africa including, population demographics, cross-reactive protection from other human or bat coronaviruses, differences in infectious disease burden and limited testing and reporting capacity [1, 2]. In HICs vaccination campaigns were rolled out rapidly and deployed from January 2021 [3]. Although the COVAX program facilitated increased access to COVID-19 vaccines globally [4], LMICs still had significant deficits in supplies, resulting in later onset of vaccination and lower vaccination rates [1–3, 5]. Mass COVID-19 vaccination campaigns in most African countries only started in 2022, a year after start of vaccination in HIC [3].

Most COVID-19 vaccines were developed, manufactured and tested in HICs [6], with reports of vaccine efficacy (VE) generated mostly also in HICs [7, 8]. Given that VE for vaccines against several diseases, including tuberculosis, rotavirus, yellow fever and malaria have been shown to be lower in LMICs compared to HICs [9], it is imperative that VE of all new vaccines produced in HICs are assessed in LMICs as well. Differences in host genetics, early years environments including exposure to previous infections and co-infections with other pathogens likely contribute significantly to the geographical differences in reported vaccine efficacy [9–11]. However, further studies are needed to clearly define the causes of altered VE in LMICs. COVID-19 vaccines were not extensively trialled in LMICs and, very little data on vaccine responses are available from countries in Africa.

To date, no formal direct correlates of protection have been agreed for COVID-19 vaccines [12]. The best proxy for vaccine protection are the inhibitory concentration of neutralizing antibodies (NAb), reported as neutralizing antibody titres (NAbTs) against individual SARS-CoV-2 variants [12–14]. It should be noted that NAb correlates with vaccine protection were demonstrated at the onset of the pandemic when the population was mostly naïve for SARS-CoV-2, as opposed to the current era with high levels of SARS-CoV-2 exposure. Little is known about the vaccine-elicited NAb responses against COVID-19 in African populations including the interactions between waning NAb levels and sequential emerging variants over time [15]. The extent to which individuals in LMICs in Africa are protected from SARS-CoV-2 infections after vaccination is not well known [16, 17]. Studies investigating protectiveness of COVID-19 vaccines in a setting of high natural infections, as is the case in most African countries due to late start of vaccination, have been mostly performed in HICs [18]. Investigation of the immune response in African populations upon COVID-19 vaccination is particularly relevant both because new SARS-CoV-2 variants with unique genetic and antigenic properties continue to emerge, and despite limited vaccine coverage, mortality and morbidity from severe COVID-19 remains low [19].

To investigate immune responses, including NAbT following COVID-19 vaccination [20], we prospectively enrolled adults undergoing primary course COVID-19 vaccination (AZD1222, Oxford-AstraZeneca) or BNT162b2 (Pfizer-BioNTech) at health centres in Accra, Ghana. This prospective cohort study “HERITAGE”, has been designed in parallel to the LEGACY study in the UK, an entirely representative cohort of the UK clinical trial population [21]. This unique paired design between the two studies allows for in-depth exploration of the biological basis underlying differences in the vaccine responses between different geographical locations over 12 months post-first vaccine dose. Here, we report longitudinal analysis of NAbT against SARS-CoV-2 variants relevant to West Africa across the two cohorts between 2021–2022.

## Methods

### Study design and participant recruitment criteria

The HERITAGE study is a prospective cohort study based in Accra, Ghana, recruiting adults presenting to urban health centres for first dose COVID-19 vaccination from December 2021 with follow up to February 2023, in a parallel design to the UCLH-Crick LEGACY study in the UK [21] to allow for investigation into the biological basis underlying differences in vaccine response. 301 participants were recruited and sampled at baseline (before receiving the first dose of the COVID-19 vaccine, day 0) and then at approximately 1 week, 3 weeks, 6 weeks, 3 months, 6 months, and 12 months. Participants received the first dose of the COVID-19 vaccine at baseline. The median interval between the first and second dose was 28 days (IQR 28–36 days) for AZD1222 and 21 days (IQR 21–21 days) for BNT162b2. Demographic data and participant-reported COVID-19 symptoms, hospitalisation and vaccination were recorded through an interview during the baseline visit. Data on COVID-19 symptoms and hospitalisation were recorded through an interview every visit and every two weeks by phone call during the study period. Inclusion criteria were provision of signed informed consent, be of African descent, be 18 years or older and not yet vaccinated with any of the COVID-19 vaccines. In addition, participants had to be willing to take at least two doses of either AZD1222 or BNT162b2 COVID-19 vaccines, donate blood, and be sampled for SARS-CoV-2 testing at each time point, and be available and willing to adhere to scheduled visits for the follow-up period. Participants who were below 18 years of age, unwilling to complete the minimum two scheduled doses and/or adhere to scheduled visits, were excluded.

Study design and recruitment criteria for the LEGACY cohort have been published before [21–24] and are described in the Additional file: Supplementary Materials and Methods [25–29].

### Study sites for the HERITAGE study

The study was conducted within the Greater Accra Region of Ghana (Additional file: Figure S1), which is the most densely populated region in Ghana, with about 5.5 million inhabitants. The Greater Accra Region was the epicentre during the early SARS-CoV-2 outbreak and continues to record the highest reported number of COVID-19 cases, accounting for 56% of all COVID-19 cases during the first two years of the pandemic [30]. The region is integral for the continued molecular genomic surveillance of SARS-CoV-2 in Ghana [31–33].

Within the HERITAGE study there is a sub-cohort of 39 participants with autoimmune disease. All these patients were recruited from the rheumatology unit of the Korle Bu Teaching Hospital. Demographics for this sub-cohort are provided in the additional file (Additional file: Table S1). The participants in the auto-immune sub-cohort were included in the general participant analysis.

### Data collection and management

Questionnaires were initially administered using Google Forms and later replaced with Uvosyo, a clinical data management system (Yemaachi Biotechnology, Accra, Ghana). Questionnaires covered data on demographic and socioeconomic status, participant-reported clinical conditions, and symptoms, as well as SARS-CoV-2 screening tests before and after vaccination. To investigate underlying conditions participants were asked: ‘Do you have any underlying conditions?’ and given the following options: asthma, autoimmune disease, diabetes, fatty liver, heart disease/hypertension, kidney disease, sinusitis, jaundice, hepatitis, sickle cell disease, stomach ulcer, typhoid. To record COVID-19 symptoms participants were asked: ‘Do you have any of the following symptoms? Body pain, cough, dizziness, fatigue, fever, headache, loss of smell or taste, sore throat, cold, stomachache'. All participant data were anonymised (i.e. de-identified by assigning laboratory-assigned codes rather than individual names).

### Sample collection and processing

Blood samples were collected into Serum Separator Tubes, SST (Jactermac, Germany) and centrifuged at 800 ⨉ g for 10 min in a benchtop centrifuge (Four E’S Scientific, Guangzhou, China) to separate serum. Serum was stored at −80 °C. Oropharyngeal swab samples were collected into viral transport media (Sansure Biotech Inc., Hunan Province, China; Sanbao Ghana Pharmaceuticals, Tema, Ghana) and transported on ice.

### SARS-CoV-2 testing by RT-PCR

SARS-CoV-2 RNA screening by real-time reverse transcription PCR (RT-PCR) was done using the novel coronavirus (2019-nCoV) Nucleic Acid Diagnostic Kit (PCR-Fluorescence Probing) (Sansure Biotech Inc., Hunan Province, China). Detailed description of the procedure is given in the additional file (Additional file: Supplementary Materials and Methods).

### Serological analysis

High-throughput live-virus neutralisation was assessed for all samples, following previously reported methods [21]. We tested neutralisation of sera against six SARS-CoV-2 strains (Additional file: Supplementary Materials Table 1). Anti-Spike (anti-S) and anti-nucleocapsid (anti-N) antibodies (IgG) were quantified using the Roche Elecsys platform according to the manufacturer’s instructions (Roche).

**Table 1:** Socio-demographic characteristics of HERITAGE study participants

Characteristics	N (%)
**Overall**	301 (100.0)
**Vaccination Site**	
Adabraka Polyclinic	155 (51.5)
Ridge Hospital	18 (6.0)
Korle-Bu	39 (13.0)
Legon Hospital	89 (29.6)
**Age, Median (IQR)**	27 (22–36)
**Age group (years)**	
18–19	18 (6.0)
20–29	157 (52.2)
30–39	69 (22.9)
40–49	29 (9.6)
50 +	28 (9.3)
**Sex**	
Female	119 (39.5)
Male	182 (60.5)
**Name of vaccine**	
AstraZeneca	139 (46.2)
Pfizer	162 (53.8)
**Nationality**	
Ghanaian	297 (98.7)
Non-Ghanaian	4 (1.3)
**Household size, Median (IQR)**	4 (3–6)
**Household size**	
1–3 members	117 (38.9)
4–6 members	134 (44.5)
7 + members	50 (16.6)
**Occupation-Category**	
Formal	63 (20.9)
Informal	120 (39.9)
Student	97 (32.2)
Unemployed	21 (7.0)
**Average monthly income in GHC**	
0–1000	190 (63.1)
1001–2500	30 (10.0)
Above 2500	15 (5.0)
Prefer not to say	66 (21.9)
**COVID-19 test history at baseline**	
Never tested	257 (85.4)
Tested Negative	38 (12.6)
Tested positive	6 (2.0)
**Autoimmune disease**	
No	262 (87.0)
Yes	39 (13.0)
**Co-morbid disease burden excluding autoimmune disease**	
None	276 (91.7)
1 condition	20 (6.6)
2 + conditions	5 (1.7)
**Number of COVID-19 symptoms at baseline**	
None	228 (75.7)
1 symptom	61 (20.3)
2 + symptoms	12 (4.0)

### Statistical analysis

Bivariate analysis of the association between COVID-19 test results at each follow-up period and the observed socio-demographic characteristics was assessed using the Pearson’s Chi-square test for socio-demographics with categorical independent variables. For analysis of the association between COVID-19 test results and socio-demographic characteristics with continuous independent variables the Wilcoxon rank sum test was used because a normal distribution could not be assumed.

A multivariable binary logistic regression model using the repeated binary outcome approach with unstructured covariance matrix, was fitted to estimate the odds ratios of factors associated with COVID-19 positivity among the participants over the follow-up time. Variables included in the multivariable analysis were those significant at 0.100 from a simple model fitted adjusting for the time of follow-up. Multicollinearity was assessed using the variance inflation factor on the final variables. Further sub-analysis was performed to estimate the odds of COVID-19 positivity at the various follow-up periods relative to baseline among the variables considered in the final multivariable binary logistic regression model.

Analyses of live virus neutralisation was performed on data from 231 participants from whom serum samples where available for all time points. A detailed description of the analyses is provided in the supplementary data.

All statistical analysis was conducted using Stata SE version 17 (Stata Corp, College Station, TX, USA). Results were considered significant at 0.05 alpha level of significance.

### Role of the funding source

The funder of this study had no role in study design, data collection, data analysis, data interpretation, or writing of the manuscript.

## Results

### Demographic and clinical characteristics of participants

#### Baseline demographics of the HERITAGE cohort

The HERITAGE cohort (*n* = 301) had a median age of 27 years with an interquartile range of 22 to 36 years, and the majority were male (60.5%) (Table 1). Around half of the participants (139, 46.2%) received AZD1222 vaccine, whereas the other half (162, 53.8%) received BNT162b2 (Table 1). The median household size of the cohort was 4 members. Of the participants, 7.0% were unemployed, 20.9% were formally employed, 39.9% were informally employed, and 32.2% were students (Table 1). The vast majority (85.4%) had never been tested for COVID-19, while 12.6% had previously tested negative and 2.0% had previously tested positive (Table 1).

Most participants did not report any co-morbid diseases (91.7%), whereas 6.6% reported one condition and 1.7% reported two or more conditions. The most common co-morbidities reported were heart disease/hypertension, diabetes, and asthma (Additional file: Figure S1). At baseline 24.3% of the participants reported having COVID-19 symptoms at that time. The common COVID-19 symptoms reported included headache, body pain, fever, cough, and sore throat (Additional file: Figure S1).

#### COVID-19 positivity among the HERITAGE cohort

**Fig. 1:**
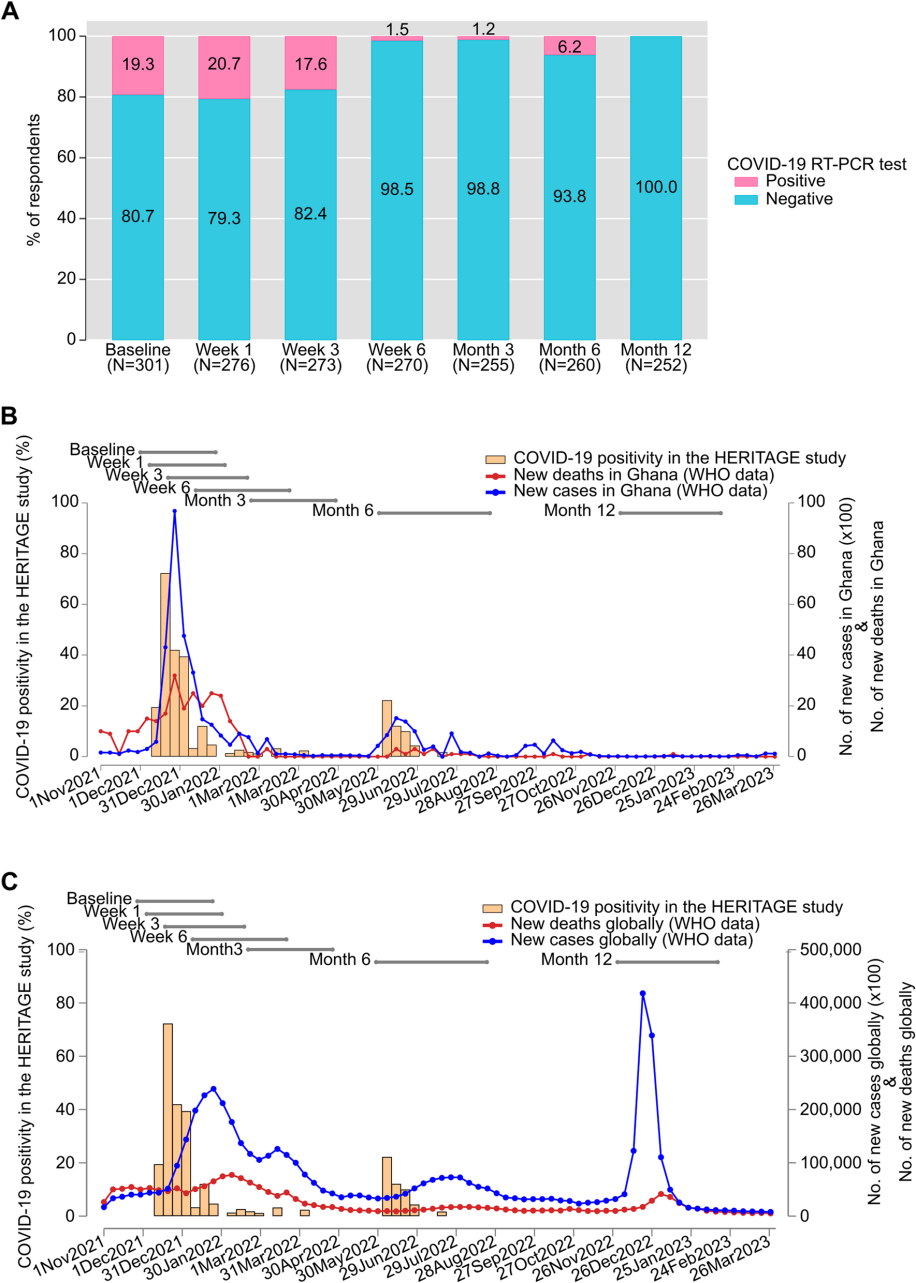
COVID-19 positivity trends in the HERITAGE study. **A** Percentage of COVID-19 positive participants at each time point in the HERITAGE study, determined by RT-PCR. **B** Weekly percentage of COVID-19 positive participants in the HERITAGE study compared to new COVID-19 cases and COVID-19 deaths in Ghana. **C** Weekly percentage of COVID-19 positive participants in the HERITAGE study compared to new COVID-19 cases and COVID-19 deaths globally

At the beginning of the study, COVID-19 positivity as determined by RT-PCR, was about 20% and this reduced rapidly during the first weeks of the study (Fig. 1A). The rates of COVID-19 positivity in the HERITAGE cohort correlated well with surveillance data of COVID-19 in Ghana, showing a peak around December 2021 and June 2022 (Fig. 1B). Global COVID-19 positivity trends during the same period peaked in January 2022 and December 2022 (Fig. 1C). Interestingly, from the 58 participants who tested positive for COVID-19 at baseline only 17 (29.3%) reported COVID-19 symptoms (Additional file: Table S2). This suggests asymptomatic COVID-19 in 70.7% (41/58) of the participants who tested positive for COVID-19 at baseline.

**Fig. 2:**
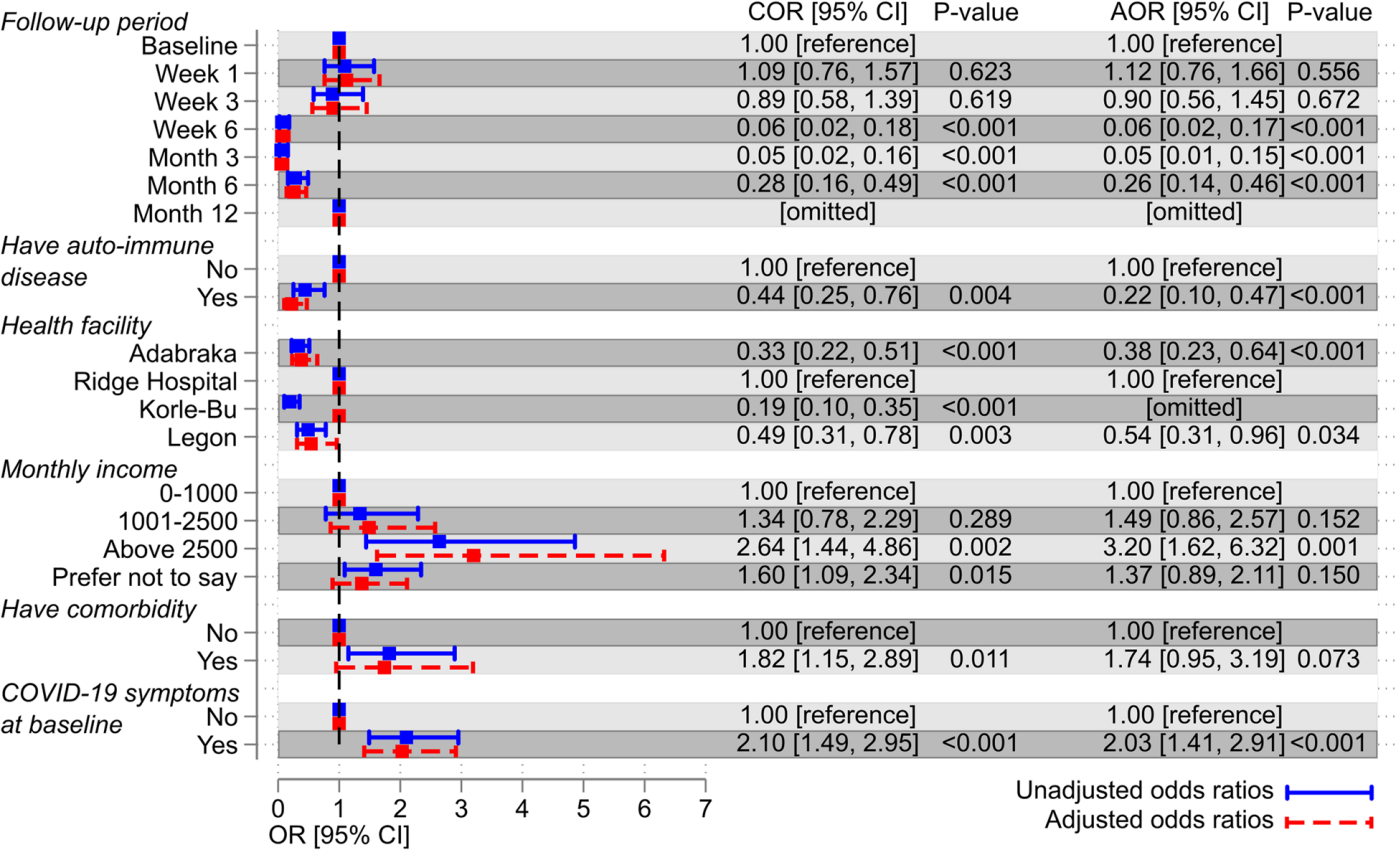
Factors associated with COVID-19 positivity. Forest plot of adjusted (red dashed line) and unadjusted (blue solid line) odds ratios for COVID-19 positivity among HERITAGE study participants over the follow-up time based on a multivariable binary logistic regression model

We found that at baseline, a monthly income of above GHC2500 (~ $400) (*p* = 0.011) and two or more COVID-19 symptoms reported (*p* = 0.023), were significantly associated with SARS-CoV-2 positivity (Additional file: Table S2). A binary logistic regression model further showed that the odds of COVID-19 positivity during the whole 12-month study period were increased among those earning above GHC2500 per month compared to those earning less than GHC1000 per month (adjusted odds ratio, AOR: 3.20, CI: 1.62–6.32, *p* = 0.001) (Fig. 2). The model also indicated that the odds of COVID-19 positivity was 78% less among those with auto-immune disease (AOR: 0.22, CI: 0.10–0.47, *p* < 0.001) (Fig. 2). Finally, the odds of COVID-19 positivity were significantly reduced starting from week 6 (AOR: 0.06, CI: 0.020–01.17, *p* < 0.001) (Fig. 2).

#### High anti-Nucleocapsid and anti-Spike antibody titres at baseline in the HERITAGE cohort

**Fig. 3:**
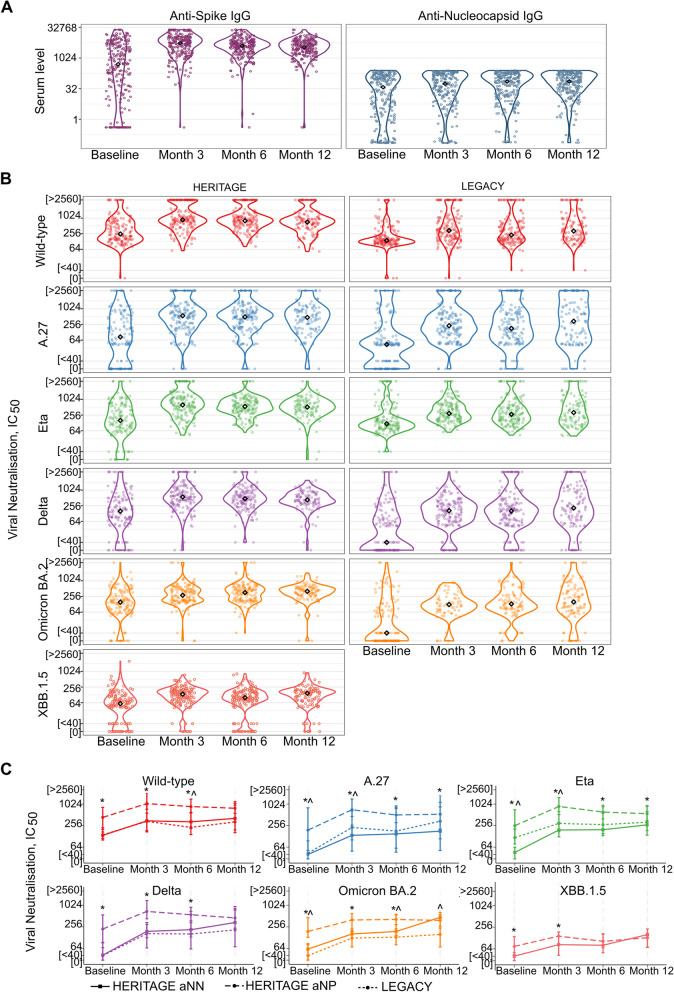
Serological analyses of HERITAGE plasma samples and comparison with LEGACY showing increased NAbTs after vaccination. **A** Violin plots depicting anti-Spike and anti-Nucleocapsid levels at baseline and at 3 months, 6 months and 12 months after the start of the study, *n* = 231. **B** Neutralising antibody titers against six different SARS-CoV-2 strains for HERITAGE, Ghana (left) and LEGACY, UK (right). Number of participants for each cohort baseline (*n* = 142), month 3 (*n* = 156), month 6 (*n* = 154), month 12 (*n* = 109) (Additional file: Table S9). **C** Median NAbTs for HERITAGE samples subdivided into a group that is COVID-19 naïve at baseline (anti-N negative, aNN), and a group this is previously exposed to COVID-19 (anti-N positive at baseline, aNP) and matched LEGACY samples. Baseline: aNN (*n* = 36), aNP (*n* = 106), LEGACY (*n* = 142), Month 3: aNN (*n* = 36), aNP (*n* = 105), LEGACY (*n* = 156), Month 6: aNN (*n* = 35), aNP (*n* = 104), LEGACY (*n* = 154), Month 12 aNN (*n* = 36), aNP (*n* = 106), LEGACY (*n* = 108). Wilcoxon rank-sum test of equality of medians was used to compare NABTs between two different groups. ^ indicates statistically significant (*p* < 0.05) difference between LEGACY and aNN. * indicates statistically significant (*p* < 0.05) difference between aNP and aNN

Longitudinal serological titres were obtained from 231/301 (76.7%) participants (Additional file: Table S3). At baseline most participants had detectable anti-Spike (anti-S) (190/231, 82.3%) and anti-Nucleocapsid IgG (anti-N) levels (186/231, 80.5%), indicative of prior exposure to SARS-CoV-2 (Fig. 3A, Additional file: Table S4). As expected, median anti-S levels further increased upon vaccination, from 1284.5 (IQR 183.0–5470.2) at baseline to 5548.0 (IQR 1859.0–10,660.2) at month three (Fig. 3A, Additional file: Table S4).

A small group of participants were negative for anti-N (45/231, 19.5%) and anti-S (41/231, 17.7%) at baseline (Fig. 3A). The anti-N negative (aNN) participants were more likely to test positive for SARS-COV-2 by PCR at week 1 (p < 0.001) and month 6 compared (p = 0.005) to those who were anti-N positive (aNP) at baseline (Additional file: Figure S2).

#### NAbTs against A.27, Omicron BA.2, and XBB.1.5 show no waning during 12 months after vaccination

NAbTs against six different SARS-CoV-2 strains were determined using a high-throughput live-virus neutralisation assay (Additional file, Table S5). The median titres (IQR) at baseline were 307.5 (159.6–808.5), 134.2 (41.8–633.2), 206.7 (82.1–589.7), 189.2 (85.9–509.9), 175.0 (86.1–397.9) and, 65.7 (10.0–117.6), for Wild-type, A.27, Eta, Delta, BA.2, and XBB.1.5 respectively (Additional file: Table S5). NAbTs were boosted at three months after the first dose of vaccination (Wild-type: Fold change (FC) relative to baseline 3.07, *p* < 0.001, A.27: FC 4.59 *p* < 0.001, Eta: FC 3.49 *p* < 0.001, Delta: FC 2.97 *p* < 0.001, BA.2: FC 1.89 *p* < 0.001, XBB.1.5: FC 1.93 *p* < 0.001) (Fig. 3B, Table 2, Additional file: Figure S2). Over the 12-month study period NAbTs remained high, although there was a slight decrease in NAbTs against Wild-type (FC 0.77, *p* < 0.001), Eta (FC 0.72, *p* < 0.001) and Delta (FC 0.76, *p* < 0.01) between month 3 and month 12 (Fig. 3B, Table 2, Additional file: Figure S2), consistent with waning expected over time. NAbTs against A.27 (FC 0.85, *p* = ns), BA.2 (FC 1.11, *p* = ns) and XBB.1.5 (FC 1.06, *p* = ns) did not show waning (Fig. 3B, Table 2, Additional file: Figure S2). No significant differences in NAbTs were found between participants vaccinated with AZD1222 or BNT162b2 (Additional file: Figure S2).

**Table 2:** Fold changes between times points across all variants for HERITAGE and LEGACY

	**Total HERITAGE**	**HERITAGE aNN**	**HERITAGE aNP**	**LEGACY**
	**Fold change [95%CI]**	**Fold change [95%CI]**	**Fold change [95%CI]**	**Fold change [95%CI]**
**Wild-type**				
Month 3 (ref: Baseline)	3.07 [2.23–3.96] ***	2.88 [1.74–4.04] ***	2.53 [1.87–3.12] ***	2.39 [1.88–2.83] ***
Month 6 (ref: Baseline)	2.59 [1.91–3.21] ***	2.37 [1.80–3.76] ***	2.00 [1.51–2.49] ***	1.62 [1.21–2.20] ***
Month 12 (ref: Baseline)	2.36 [1.74–2.92] ***	2.67 [1.70–4.26] ***	1.78 [1.34–2.15] ***	2.30 [1.72–3.33] ***
Month 6 (ref: Month 3)	0.84 [0.70–0.99] *	0.82 [0.61–1.57]	0.79 [0.68–0.98] *	0.68 [0.49–0.97] *
Month 12 (ref: Month 3)	0.77 [0.63–0.90] ***	0.93 [0.59–1.73]	0.71 [0.61–0.83] ***	0.96 [0.70–1.47]
Month 12 (ref: Month 6)	0.91 [0.78–1.04]	1.13 [0.63–1.71]	0.89 [0.75–1.01]	1.42 [0.95–2.30]
**A.27**				
Month 3 (ref: Baseline)	4.59 [3.32–8.47] ***	29.22 [20.12–35.59] ***	3.81 [2.24–4.67] ***	5.26 [3.99–7.69] ***
Month 6 (ref: Baseline)	3.81 [2.88–6.76] ***	32.52 [15.18–56.53] ***	3.00 [1.71–3.88] ***	4.17 [3.20–7.70] ***
Month 12 (ref: Baseline)	3.92 [2.80–6.93] ***	35.78 [29.48–54.06] ***	3.01 [1.76–3.83] ***	7.85 [3.50–15.36] ***
Month 6 (ref: Month 3)	0.83 [0.68–1.03]	1.11 [0.49–2.50]	0.79 [0.64–0.97] *	0.79 [0.50–1.59]
Month 12 (ref: Month 3)	0.85 [0.65–1.05]	1.22 [0.93–2.28]	0.79 [0.67–0.97] *	1.49 [0.61–3.42]
Month 12 (ref: Month 6)	1.03 [0.81–1.20]	1.10 [0.58–2.86]	1.00 [0.80–1.28]	1.88 [0.69–3.97]
**Eta**				
Month 3 (ref: Baseline)	3.49 [2.72–4.69] ***	4.78 [3.10–24.50] ***	3.13 [2.13–4.13] ***	2.50 [1.96–3.26] ***
Month 6 (ref: Baseline)	2.69 [2.17–3.55] ***	5.15 [3.45–28.34] ***	2.27 [1.61–2.82] ***	2.31 [1.76–2.85] ***
Month 12 (ref: Baseline)	2.51 [2.04–3.15] ***	6.66 [4.50–33.21] ***	1.96 [1.38–2.44] ***	2.71 [1.68–4.17] ***
Month 6 (ref: Month 3)	0.77 [0.62–0.99] *	1.08 [0.74–2.08]	0.73 [0.59–0.89] ***	0.92 [0.68–1.18]
Month 12 (ref: Month 3)	0.72 [0.57–0.87] ***	1.40 [1.01–2.21] *	0.63 [0.52–0.77] ***	1.08 [0.66–1.69]
Month 12 (ref: Month 6)	0.93 [0.77–1.08]	1.29 [0.69–1.98]	0.86 [0.76–1.01]	1.17 [0.74–1.89]
**Delta**				
Month 3 (ref: Baseline)	2.97 [2.42–3.67] ***	4.60 [3.18–23.85] ***	2.60 [1.68–3.31] ***	16.51 [3.00–24.27] ***
Month 6 (ref: Baseline)	2.53 [2.08–3.14] ***	5.12 [3.38–22.88] ***	2.08 [1.37–2.64] ***	16.18 [3.17–25.71] ***
Month 12 (ref: Baseline)	2.26 [1.89–2.75] ***	7.80 [5.63–34.61] ***	1.76 [1.19–2.22] ***	20.98 [3.93–36.60] ***
Month 6 (ref: Month 3)	0.85 [0.72–1.02]	1.11 [0.70–1.87]	0.80 [0.67–0.96] *	0.98 [0.72–1.43]
Month 12 (ref: Month 3)	0.76 [0.66–0.89] **	1.69 [1.18–2.25] **	0.68 [0.59–0.81] ***	1.27 [0.87–2.18]
Month 12 (ref: Month 6)	0.89 [0.78–1.02]	1.52 [0.92–2.11]	0.85 [0.74–0.99] *	1.30 [0.90–2.08]
**Omicron BA.2**				
Month 3 (ref: Baseline)	1.89 [1.49–2.28] ***	2.61 [2.11–3.17] ***	1.93 [1.34–2.18] ***	12.42 [10.19–27.27] ***
Month 6 (ref: Baseline)	2.08 [1.87–2.49] ***	3.25 [2.46–5.16] ***	1.93 [1.38–2.26] ***	13.15 [10.16–30.74] ***
Month 12 (ref: Baseline)	2.10 [1.86–2.49] ***	6.37 [4.04–8.84] ***	1.81 [1.31–2.13] ***	15.70 [10.68–46.25] ***
Month 6 (ref: Month 3)	1.10 [0.95–1.43]	1.25 [0.99–1.91]	1.00 [0.89–1.20]	1.06 [0.76–1.51]
Month 12 (ref: Month 3)	1.11 [0.94–1.44]	2.44 [1.61–3.31] ***	0.94 [0.85–1.14]	1.26 [0.82–2.24]
Month 12 (ref: Month 6)	1.01 [0.86–1.16]	1.96 [0.98–2.85]	0.93 [0.81–1.12]	1.19 [0.76–2.19]
**XBB.1.5**				
Month 3 (ref: Baseline)	1.93 [1.65–2.45] ***	8.37 [1.73–11.02] ***	1.93 [1.53–2.28] ***	
Month 6 (ref: Baseline)	1.55 [1.34–1.87] ***	8.07 [1.74–11.83] ***	1.38 [1.15–1.70] ***	
Month 12 (ref: Baseline)	2.04 [1.65–2.53] ***	15.95 [2.98–17.36] ***	1.75 [1.30–2.12] ***	
Month 6 (ref: Month 3)	0.80 [0.67–0.94] **	0.96 [0.66–1.60]	0.72 [0.63–0.90] **	
Month 12 (ref: Month 3)	1.06 [0.81–1.27]	1.91 [1.16–2.69] *	0.90 [0.70–1.13]	
Month 12 (ref: Month 6)	1.32 [1.05–1.55] *	1.98 [1.13–2.47] *	1.26 [0.96–1.49]	

Fold changes in neutralising antibody titres (NAbTs) are calculated between two time points for each cohort. Statistical significance indicates that NAbTs for the later time point are significantly different from NAbTs for the reference time point. *P-*values are calculated using a pairwise test of equality of median using 1,000 bootstrapped standard errors. aNN: anti-N negative at baseline. aNP: anti-N positive at baseline. CI: confidence interval. *P*-value notation: *p* < 0.05*; p < 0.01**; *p* < 0.001***

### Different kinetics in NAbTs between participants with and without prior SARS-CoV-2 exposure

Different NAb kinetics have been reported for COVID-19 naïve vaccinees versus those who had previously been infected with SARS-CoV-2 [34]. We therefore investigated NAbTs in the anti-Nucleocapsid negative (aNN) and anti-Nucleocapsid positive (aNP) subgroups of the HERITAGE cohort. As expected, NAbTs were lower in aNN compared to aNP (median NAbT for Wildtype: 143.7 [IQR 101.8–177.8] vs 437.2 [202.5–919.9] *p* < 0.0001, for A.27: 5.0 [5.0–5.0] vs 191.5 [57.5–784.1] *p* < 0.0001, for Eta: 45.7 [10.0–63.9] vs 279.2 [133.1–723.8] *p* < 0.0001, for Delta: 42.8 [5.0–70.3] vs 248.6 [133.1–616.4] *p* < 0.0001, for Omicron BA.2: 63.1 [10.0–87.3] vs 200.6 [135.5–482.1] *p* < 0.0001, for XBB.1.5: 10.0 [5.0–50.7] vs 74.9 [44.3–139.3] *p* < 0.0001) (Fig. 3C, Additional file: Figure S3 and Table S6). Interestingly, there was a difference in the NAbTs kinetics between aNN and aNP during the 12-month study period. The aNP group showed an increase in NAbTs for all six SARS-CoV-2 variants from baseline to month 3, and a decrease for Wild-type (FC 0.71, *p* < 0.001), A.27 (FC 0.79, *p* = 0.05), Eta (FC 0.63, *p* < 0.001) and Delta (FC 0.68, *p* < 0.001) between month 3 and month 12 (Fig. 3C, Table 2). The aNP group did not show waning for Omicron BA.2 (FC 0.94, ns) and XBB.1.5 (FC 0.90, ns) the two Omicron variants that were present during the time of the study (Fig. 3C, Table 2). The aNN group, on the other hand, showed an increase in NAbTs between month 3 and month 12 for Eta (FC 1.40, *p* = 0.05), Delta (FC 1.69, *p* < 0.01), Omicron BA.2 (FC 2.44, *p* < 0.001) and XBB.1.5 (FC 1.91, *p* = 0.05) and no difference for Wild-type (FC 0.93, ns) and A.27 (FC 1.22, ns) (Fig. 3C, Table 2). For Omicron BA.2 and XBB.1.5 aNN reached similar NAbTs as aNP by months 12, 402.1 [197.7–640.4] vs 362.4 [239.8–520.2] (*p* = 0.921) for Omicron BA.2 and 159.5 [71.1–228.6] vs 130.7 [70.0–173.3] (*p* = 0.294) for XBB.1.5 (Fig. 3C, Additional file: Figure S3 and Table S6).

#### NAbTs against SARS-CoV-2 are generally higher and more durable in the HERITAGE cohort (Ghana) versus the LEGACY study (UK)

We then tested for differences in both the vaccine-induced boosting and waning in our Ghanaian cohort with an age/gender/infection and vaccine history matched cohort from London (Table 3, Additional file: Table S7). LEGACY is an observational cohort investigating serological responses to COVID-19 vaccines following the UK vaccine roll out in January 2021 [21]. Samples were curated in a sequential order following vaccine dose 1 for matching, to minimise confounding effect of accumulated exposures inferred by the time-lag bias between the two cohorts (Additional file: Figure S4). At baseline, median neutralisation levels against the Delta and the Omicron BA.2 variants in LEGACY participants were < 40 or 0 respectively, in comparison with higher median neutralisation titres (NAbTs) above 40 already at baseline in the HERITAGE cohort (Fig. 3B and C). Of note, pre-existing NAbT against variants that had or were currently circulating in Ghana were detected pre-vaccine in the HERITAGE cohort, but were undetectable in the LEGACY cohort. Median NAbTs were overall higher at all timepoints tested in the HERITAGE cohort compared to the LEGACY cohort, with exception of Eta at baseline and A.27 at 12 months (Fig. 3B and C, Additional file: Table S8). Comparison of the LEGACY data to the aNN and aNP subgroups showed that, with exception for Omicron BA.2, LEGACY data showed similar kinetics to the HERITAGE aNN group (Fig. 3C). For Omicron BA.2, in the aNN subgroup from the HERITAGE cohort, NAbTs continued to increase between month 3 and month 12 (median 470.0, FC 2.44, *p* < 0.001) reaching similar levels as aNP by month 12, whereas in LEGACY NAbTs against Omicron BA.2 were maintained at a relatively low level from month 3 to month 12 (median 157.0, FC 1.26, *p* = ns) (Table 2 and Additional file: Table S9-11).

**Table 3:** Socio-demographic characteristics of matched HERITAGE and LEGACY study participants at each time point

	**Baseline N (%)**		**Month 3 N (%)**		**Month 6 N (%)**		**Month 12 N (%)**	
	**Heritage**	**Legacy**	**Heritage**	**Legacy**	**Heritage**	**Legacy**	**Heritage**	**Legacy**
**Total**	142	142	156	156	154	154	109	109
**Match type**								
Fuzzy	0 (0)	0 (0)	14 (9.0)	14 (9.0)	89 (57.8)	89 (57.8)	101 (92.7)	101 (92.7)
Matched	142 (100.0)	142 (100.0)	142 (91.0)	142 (91.0)	65 (42.2)	65 (42.2)	8 (7.3)	8 (7.3)
**Age group**								
18–30	45 (31.7)	45 (31.7)	55 (35.3)	55 (35.3)	53 (34.4)	53 (34.4)	29 (26.6)	29 (26.6)
30–40	53 (37.3)	53 (37.3)	57 (36.5)	54 (34.6)	57 (37.0)	52 (33.8)	43 (39.4)	43 (39.4)
40–50	22 (15.5)	24 (16.9)	22 (14.1)	28 (17.9)	22 (14.3)	30 (19.5)	18 (16.5)	25 (22.9)
> 50	22 (15.5)	20 (14.1)	22 (14.1)	19 (12.2)	22 (14.3)	19 (12.3)	19 (17.4)	12 (11.0)
**Sex**								
Females	93 (65.5)	93 (65.5)	93 (59.6)	93 (59.6)	92 (59.7)	92 (59.7)	71 (65.1)	71 (65.1)
Males	49 (34.5)	49 (34.5)	63 (40.4)	63 (40.4)	62 (40.3)	62 (40.3)	38 (34.9)	38 (34.9)

## Discussion

West Africa experienced strikingly lower mortality and morbidity from COVID-19 in 2020–2021 than many higher-income settings in Europe and the US, despite limited use of non-pharmaceutical interventions and delayed deployment of COVID-19 vaccines [1, 2, 5, 35]. Our comparison between parallel prospective cohorts from both Ghana and the UK provides a unique insight into the differing exposure and subsequent serological response to vaccination across these two regions.

This first analysis of our HERITAGE cohort in Ghana shows that the majority COVID-19 positive participants did not report COVID-19 symptoms, indeed suggesting more mild COVID-19 in Ghana. The high level of asymptomatic COVID-19 in our study participants at baseline, in combination with evidence of extensive, pre-vaccine, infection induced immunity in our cohort further suggests differences in both early exposure and the host response differed in Ghana compared to HIC.

We found strikingly higher NAbTs against all six SARS-CoV-2 variants tested in our Ghanaian cohort compared to our UK cohort. These titres were similarly boosted with both adenovirus-vectored and mRNA vaccination, in line with reports from other West-African countries [36]. We found minimal waning of NAbT between three and six months following dose 2 in the Ghanaian cohort. The kinetics of the NAbTs in the aNP group, followed a classical antibody peak-wane pattern of a typical memory B-cell response [34], and were strikingly higher than in the UK cohort. Rates of seroconversion following prior infection were higher in the Ghanaian cohort and associated with higher NAbT peaks and limited wane. Recent analysis of later time points from the LEGACY study showed that higher NAbTs were observed in later sARS-CoV-2 waves, especially in those with previous SARS-CoV-2 infection [37]. The high NABTs observed in HERITAGE more closely resemble the later waves in LEGACY, suggesting that earlier infection has boosted the pre-existing population immunity in the HERITAGE cohort. Our findings are further supported by another study in West-Africa showing high NAbTs and limited wane after vaccination in individuals with prior natural exposure to SARS-CoV-2 [15].

Unsurprisingly, participants who were infection naïve, as indicated by lack of anti-N IgG at baseline, had overall lower neutralisation levels than participants who were anti-N positive. In contrast to the previously infected participants, NAbTs for the infection-naïve group were maintained between month 3 and month 12 for the Wild-type, Eta, Delta and A.27 variants and continued to go up for the Omicron B.A2 and XBB.1.5 variants. The number of participants without anti-Nucleocapsid IgG decreased during this time, suggesting that the increased NAbTs are a result of breakthrough infections. At 12 months both the aNN and aNP groups reached similar levels of NAbTs against Omicron B.A2 and XBB.1.5. This is in line with other studies where there were no differences in NAbTs between infected-then-vaccinated and vaccinated-then-infected when participants received two doses of a COVID-19 vaccine [34]. Interestingly, NAbTs against non-Omicron variants did not respond as strongly as the NAbTs against the Omicron variants. This suggests that natural infection with Omicron has a more pronounced effect on Omicron specific antibodies and only has a moderate effect on the other NAbs generated after vaccination. Future research is needed to further study how breakthrough infections shape the serological immune response, especially considering immune imprinting by vaccination.

We found an intriguing relationship between COVID-19 positivity at baseline and the highest income groups (above GHC2500), in contrast to data from HIC where COVID-19 positivity and income are most commonly negatively correlated [38]. A potential explanation could be that many participants in the lower income groups had professions like ‘trader’ that are practised in the outside and without intensive contacts with colleagues, in contrast to ‘office worker’ or ‘teacher’ found in the higher income groups. However, the number of participants in the highest income group was very small (above GHC5000, 2/301, 0.7%) and an income between GHC2500 – GHC5000 as reported by 13 participants (4.3%) is around the monthly living wage required for a basic but decent living standard, which has been set at GHC2,922 for Ghana in 2023 [39].

One of the unique features of the HERITAGE study is its comparable design to the LEGACY study. Here, we report how infection naïve vaccine recipients in Ghana show similar NAbTs kinetics as vaccine recipients in the UK for Wild-type, A.27, Eta and Delta SARS-CoV-2 variants. Interestingly, while kinetics were similar, NAbTs for A.27 and Eta at baseline and at month three were lower in the aNN HERITAGE group compared to LEGACY. These differences in pre-exposure NAbTs between the two geographically different cohorts might provide insights into the emergence of the A.27 and Eta variants in West Africa [40, 41].

Differences in the kinetics of NAbTs between LEGACY and the HERITAGE aNN group were only observed for the Omicron BA.2 variant, which was dominant during the time of HERITAGE but had not emerged during the LEGACY study period. This suggests that environmental factors, including SARS-CoV-2 infection rate and timing of the vaccination, may contribute to the differences observed between the two studies. However, further research is required to fully compare the immune response between vaccine recipients in geographically different areas, as we were not able to fully control for potential confounding factors in this analysis, including differences in seasonal and bat coronavirus exposure, nutritional status and host immune polymorphisms that might also contribute to the observed difference between the two cohorts.

While our study is comprehensive in comparing the vaccination response over twelve months in healthy adults between West Africa and London, our study conclusions are primarily limited by time-lag between deployment of vaccination in London and West Africa of approximately 12 months. Delayed deployment of COVID-19 vaccines to West Africa meant that participants in the different regions were exposed to different circulating SARS-CoV-2 variants prior to vaccination. Sampling in the LEGACY cohort was established in January 2021, while recruitment for the HERITAGE cohort was done between December 2021 and January 2022. Delta infections had peaked in Ghana in mid-2021, and Omicron was the most common circulating variant during HERITAGE recruitment [31, 42], in contrast with LEGACY where, Delta only contributed to a small proportion of all COVID-19 cases and Omicron was not reported before November 2021 [43, 44]. Additionally, participants in LEGACY received a third dose of BNT162b2 between six and twelve months on the UK prime-boost vaccine schedule, in contrast with HERITAGE where only two doses were deployed.

The HERITAGE study, with both SARS-CoV-2 exposed and naive participants at baseline, in combination with high SARS-CoV-2 exposure during the duration of the study, provides a good platform for investigating immune protection acquired from a combination of vaccination and infection, known as hybrid immunity. Hybrid immunity has been shown to increase NAb breadth and potency, through a mechanism driven by memory B cells [45]. Upon re-call of the memory B cells they are able to produce NAb with neutralizing breadth against many SARS-CoV-2 variants, including those with immune evasive properties like Omicron [45]. However, accumulation of T cells with preserved recognition of highly mutated SARS-CoV-2 variants has also been reported [46]. Further research is needed to unravel how natural exposure and vaccination affect the cellular immune response, and how this might differ between geographic locations.

## Conclusions

Two dose vaccination in healthy adults following two years of community exposure to COVID-19 generates durable and broad neutralising antibodies against future variants. Our study highlights the importance of considering local population exposure to an emerging pathogen in the design and deployment of vaccination to healthy adults.

## Supplementary Information

Additional file1 Figure S1: Location of HERITAGE study centres and baseline co-morbidities and COVID-19 symptoms. Figure S2: PCR positivity and neutralising antibody titres in HERITAGE. Figure S3: Neutralising antibody titres in SARS-CoV-2 exposed and naïve HERITAGE participants. Figure S4: Matching of HERITAGE and LEGACY samples. Table S1: Socio-demographic characteristics of study participants with auto-immune disease. Table S2: Bivariate association between socio-demographic characteristic of study participants and COVID-19 positivity. Table S3: Socio-demographics of participants included in the serological analyses. Table S4: Anti-N and anti-S positivity and values during the HERITAGE study period. Table S5: Neutralising antibody titres during the HERITAGE study period. Table S6: Comparison of neutralisation level of Heritage aNN, and Heritage aNP samples at the different timepoints. Table S7: Demographics for matched HERITAGE and LEGACY participants. Table S8: Comparison of neutralisation level of matched sample between HERITAGE and LEAGCY. Table S9: Comparison of neutralisation levels of matched samples between the aNN subgroup in HERITAGE and LEGACY. Table S10: Comparison of neutralisation level of matched samples between Heritage aNN, and Heritage aNP Table S11: Fold median over time for matched samples between matched HERITAGE aNN, HERITAGE aNP and LEGACY samples. Supplementary materials and methods. Supplementary Materials Table 1. References

## Data Availability

Additional data is available in the Supplementary Information. De-identified participant data and code are available on reasonable request and with completion of a signed data access agreement form upon contacting the corresponding author.

## Abbreviations

anti-S: Anti-Spike
anti-N: Anti-nucleocapsid
aNN: Anti-N negative
aNP: Anti-N positive
FC: Fold change
HICs: High-income countries
HRA: Human Research Authority
IQR: Interquartile range
LMICs: Low-and middle-income countries
NAb: Neutralizing antibodies
NABTs: Neutralising antibody titres
REC: Research and Ethics committee
RT-PCR: Real-time reverse transcription PCR
VE: Vaccine efficacy
