# Supplementary material for: Adults in Ghana generate higher and more durable neutralising antibody titres following primary course COVID-19 vaccination than matched UK adults: The HERITAGE Study
Source: BMC Med. 2025 May 28;23:312. doi: 10.1186/s12916-025-04157-0 (PMC12121195; doi:10.1186/s12916-025-04157-0)
Supplement: Supplementary file 1 — Additional file1 Figure S1: Location of HERITAGE study centres and baseline co-morbidities and COVID-19 symptoms. Figure S2: PCR positivity and neutralising antibody titres in HERITAGE. Figure S3: Neutralising antibody titres in SARS-CoV-2 exposed and naïve HERITAGE participants. Figure S4: Matching of HERITAGE and LEGACY samples. Table S1: Socio-demographic characteristics of study participants with auto-immune disease. Table S2: Bivariate association between socio-demographic characteristic of study participants and COVID-19 positivity. Table S3: Socio-demographics of participants included in the serological analyses. Table S4: Anti-N and anti-S positivity and values during the HERITAGE study period. Table S5: Neutralising antibody titres during the HERITAGE study period. Table S6: Comparison of neutralisation level of Heritage aNN, and Heritage aNP samples at the different timepoints. Table S7: Demographics for matched HERITAGE and LEGACY participants. Table S8: Comparison of neutralisation level of matched sample between HERITAGE and LEAGCY. Table S9: Comparison of neutralisation levels of matched samples between the aNN subgroup in HERITAGE and LEGACY. Table S10: Comparison of neutralisation level of matched samples between Heritage aNN, and Heritage aNP Table S11: Fold median over time for matched samples between matched HERITAGE aNN, HERITAGE aNP and LEGACY samples. Supplementary materials and methods. Supplementary Materials Table 1. References [file 12916_2025_4157_MOESM1_ESM.pdf]

Additional data for

Adults in Ghana generate higher and more durable neutralising antibody titres following primary course COVID-19 vaccination than matched UK adults: The HERITAGE Study

Authors

Eliza Mari Kwesi-Maliepaard[1], Yakubu Alhassan[1,2], Emmanuel K. Quaye[1], Vera M. Kotey[1], Aisha M. Mohammed[1], Seth Agyemang[1], Adelaide K. Sromani[1], Stephanie Darko[1], Erica Buadii[1], Randy Tackie[1], Harry Akligoh[1], Barikisu Ibrahim[1], David Hutchful[1], Lily Paemka[1], Emmanuella Amoako[1], Joyce M. Ngoi[1,3], Aida Manu[1] on behalf of the HERITAGE study team[1], David Greenwood[4], Edward J. Carr[4,5], Mary Y. Wu[4], David L.V. Bauer[4], Emma C. Wall[4,5,6,7], on behalf of the Crick Legacy Consortium[4], Dzifa Dey[8], Abdul Razak Quao[9], Akosua Ayisi[10], Kwame Amponsa-Achiano[10], Franklin Asiedu Bekoe[10], Gordon Awandare[3], Peter K. Quashie[3], Yaw Bediako[1,3]

Affiliations

1. Yemaachi Biotech, Accra, Ghana
2. Department of Biostatistics, School of Public Health, University of Ghana, Accra, Ghana
3. West African Centre for Cell Biology of Infectious Pathogens (WACCBIP), University of Ghana, Accra, Ghana
4. The Francis Crick Institute, London, UK
5. University College London, Gower St, London, UK
6. National Institute for Health Research (NIHR) University College London Hospitals (UCLH) Biomedical Research Centre, London, UK
7. NIHR UCLH Clinical Research Facility, London, UK
8. University of Ghana Medical School, Korle Bu Teaching Hospital, Accra, Ghana
9. Adabraka Polyclinic, Accra, Ghana
10. Ghana Health Service, Accra, Ghana

Correspondence to: Dr. Yaw Bediako, Yemaachi Biotech, Accra, Ghana. Email: yaw@yemaachi.com. Telephone:+233 50 252 8528

Contents

Figure S1: Location of HERITAGE study centres and baseline co-morbidities and COVID-19 symptoms……………………..……..2

Figure S2: PCR positivity and neutralising antibody titres in HERITAGE ………………………………………...…………..3

Figure S3: Neutralising antibody titres in SARS-CoV-2 exposed and naïve HERITAGE participants………………………………4

Figure S4: Matching of HERITAGE and LEGACY samples…………………………………………………….……………..5

Table S1: Socio-demographic characteristics of study participants with auto-immune disease……………………….…………………..6

Table S2: Bivariate association between socio-demographic characteristic of study participants and COVID-19 positivity……….7

Table S3: Socio-demographics of participants included in the serological analyses……………………..…………………..…......9

Table S4: Anti-N and anti-S positivity and values during the HERITAGE study period……………………………….…...…......11

Table S5: Neutralising antibody titres during the HERITAGE study period……………………………………..……………12

Table S6: Comparison of neutralisation level of Heritage aNN, and Heritage aNP samples at the different timepoints……….......13

Table S7: Demographics for matched HERITAGE and LEGACY participants …………………………………………………14

Table S8: Comparison of neutralisation levels of matched samples between HERITAGE and LEGACY……………………........15

Table S9: Comparison of neutralisation levels of matched samples between the aNN subgroup in HERITAGE and LEGACY........16

Table S10: Comparison of neutralisation level of matched samples between Heritage aNN, and Heritage aNP …………….........17

Table S11: Fold median over time for matched samples between matched HERITAGE aNN, HERITAGE aNP and LEGACY samples………......18

Supplementary materials and methods……………………...………………………………………………………….....19

Supplementary Materials Table 1: Summary of SARS-CoV-2 VOC/VOI used, their mutational profile and isolate source…….21

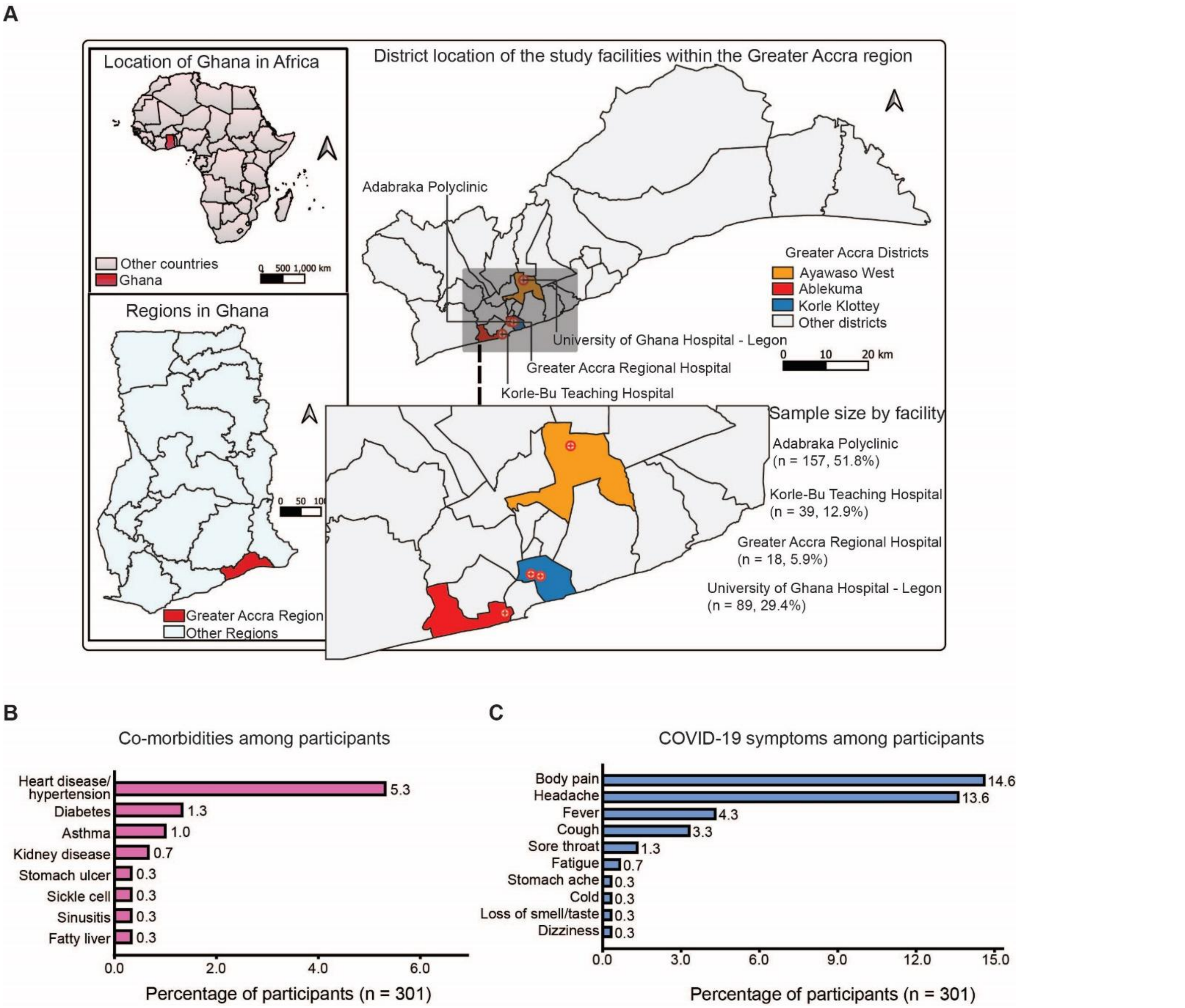

Figure S1: Location of HERITAGE study centres and baseline co-morbidities and COVID-19 symptoms. A) Location of HERITAGE study centres within the Greater Accra Region in Ghana. B-C) Frequency of co-morbidities (B) and COVID-19 symptoms (C) among study participants (n = 301)

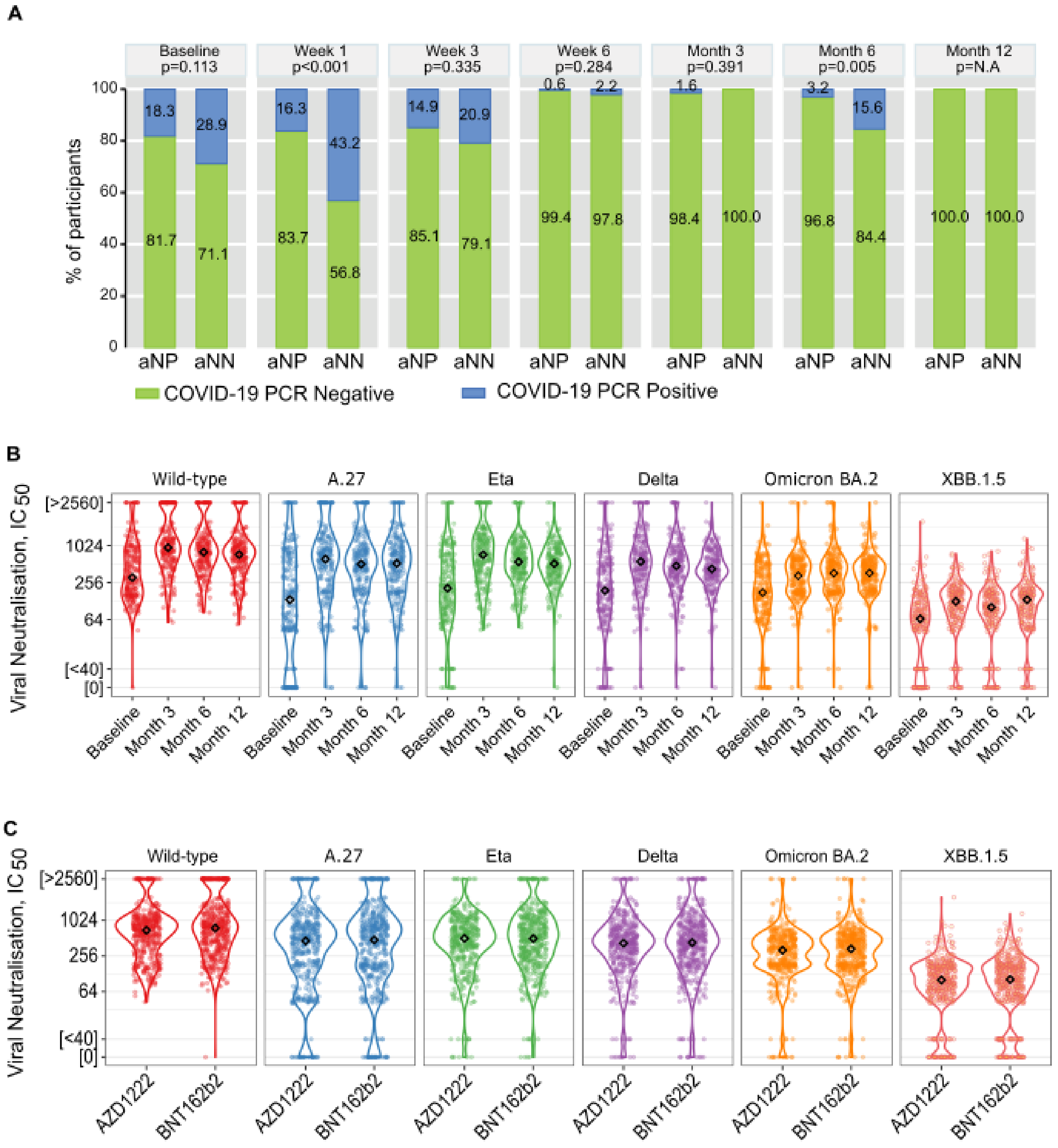

Figure S2: PCR positivity and neutralising antibody titres in HERITAGE. A) COVID-19 positivity, confirmed by qRT-PCR, in the aNN and aNP subgroups at all time points. B) Neutralising antibody titers against SARS-CoV-2 strains in HERITAGE (n=231 for each time point). C) Neutralising antibody titers against SARS-CoV-2 strains in participants vaccinated with AZD1222 and BNT126b2 for all timepoints combined (AZD1222: n = 432 for all strains, BNT162b2: n = 489 (Wild-type) n = 490 (Delta and XBB.1.5) and n = 491 (A.27, Eta and Omicron BA.2)).

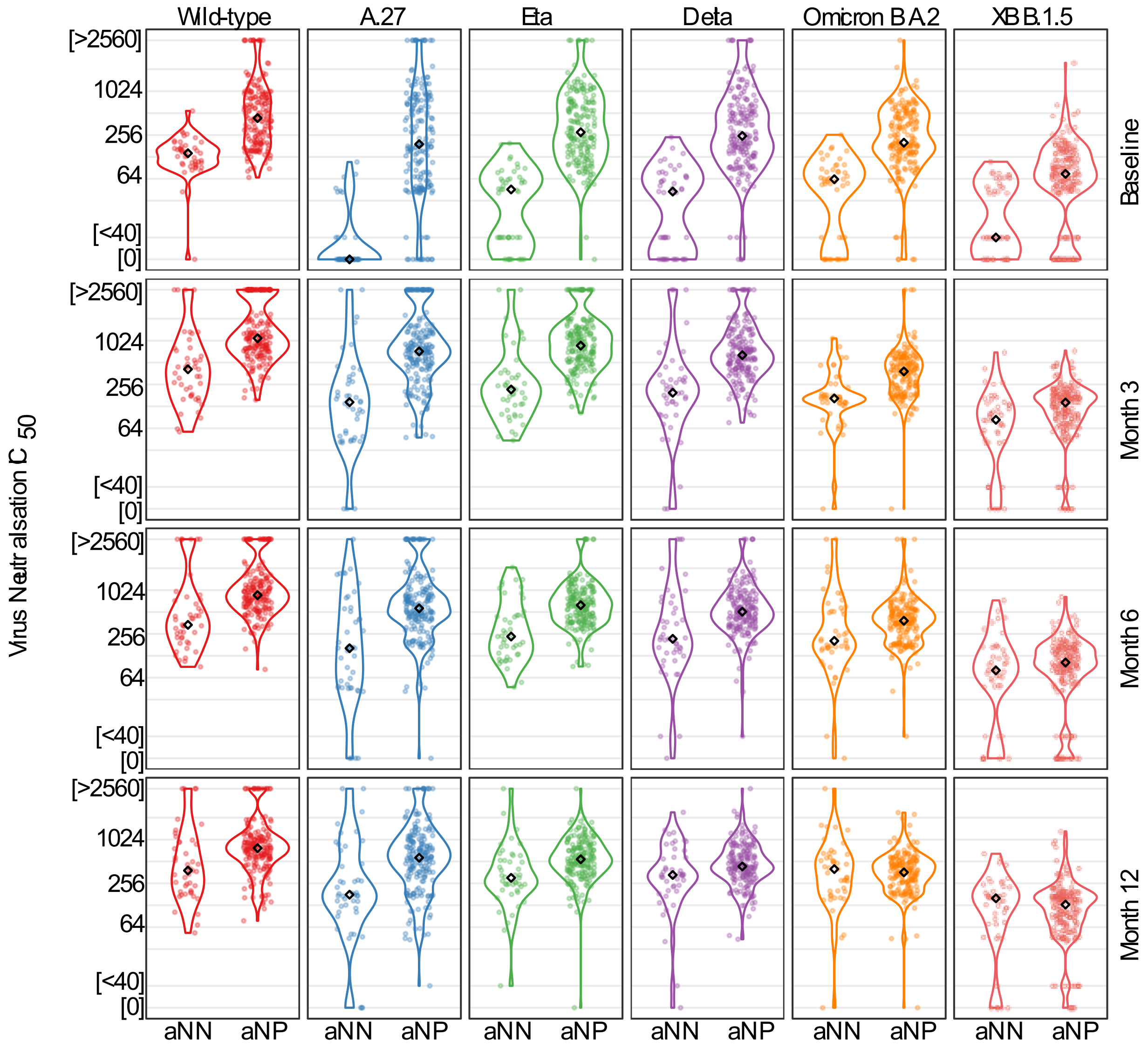

Figure S3: Neutralising antibody titres in SARS-CoV-2 exposed and naïve HERITAGE participants. Neutralising antibody titres against six SARS-CoV-2 strains for previously exposed (anti-Nucleocapsid IgG positive at baseline, aNP) and SARS-CoV-2 naïve participants (anti-Nucleocapsid IgG negative at baseline, aNN).

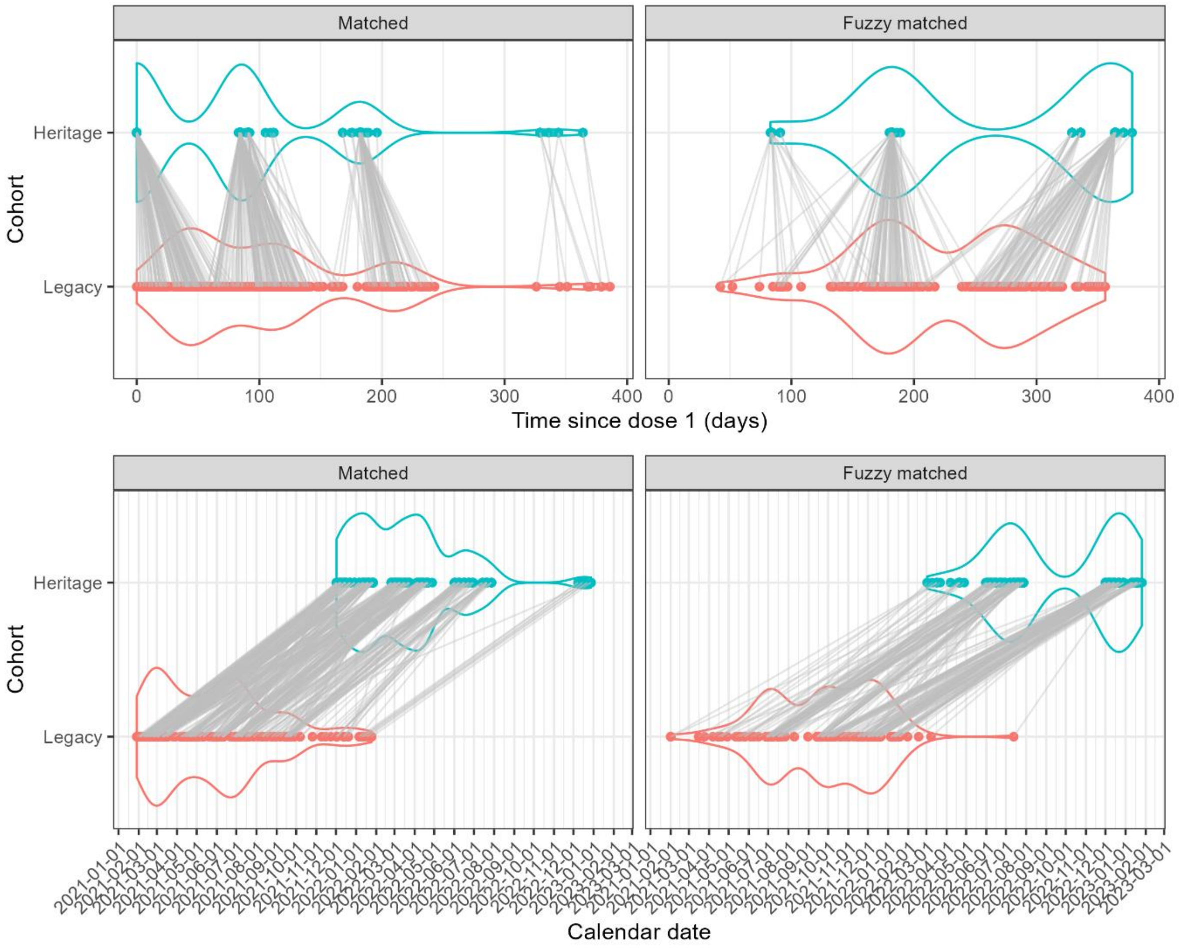

Figure S4: Comparison of matched samples between HERITAGE and LEGACY for time since dose 1 and calendar date. HERITAGE samples are depicted in blue and connected to their matched LEGCAY samples (in pink). One HERITAGE sample is matched with one LEGACY sample that is the most similar based on age, sex, vaccine type, the number of vaccine doses, and the time elapsed since the second dose, this resulted in 357 pairs of matched samples (left). The matching process was repeated with the remaining samples, but with a less strict criterion (fuzzy matched) resulting in an additional 204 pairs of samples (right).

Table S1

Socio-demographic characteristics of study participants with auto-immune disease,

**Overall**	**39 (100.0)**
**Vaccination Site**	
Korle-Bu	39 (100.0)
**Age, Median (IQR)**	35 (31-43)
**Age group**	
18-19	1 (2.6)
20-29	7 (17.9)
30-39	17 (43.6)
40-49	8 (20.5)
50+	6 (15.4)
**Sex**	
Female	37 (94.9)
Male	2 (5.1)
**Name of vaccine**	
AstraZeneca	25 (64.1)
Pfizer	14 (35.9)
**Nationality**	
Ghanaian	39 (100.0)
**Household size, median (IQR)**	5 (4-6)
**Household size**	
1-3 members	7 (17.9)
4-6 members	23 (59.0)
7+ members	9 (23.1)
**Occupation-Category**	
Formal	13 (33.3)
Informal	23 (59.0)
Student	3 (7.7)
**Average monthly income in GHC**	
0-1000	29 (74.4)
1001-2500	5 (12.8)
Above 2500	5 (12.8)
**COVID-19 test history at baseline**	
Never tested	30 (76.9)
Tested Negative	6 (15.4)
Tested positive	3 (7.7)
**Autoimmune disease**	
Yes	39 (100.0)
**Disease burden excluding auto-immune disease**	
None	33 (84.6)
1 condition	4 (10.3)
2+ conditions	2 (5.1)
**Number of COVID-19 symptoms at baseline**	
None	8 (20.5)
1 symptom	31 (79.5)

Table S2
Bivariate association between socio-demographic characteristic of study participants at baseline and COVID-19 test positivity at week 1, 3, 6, 12 and month 6 after COVID-19 vaccination.

	Follow-up period											
	**Baseline (N=301)**		**Week 1 (N=276)**		**Week 3 (N=273)**		**Week 6 (N=270)**		**Week 12 (N=255)**		**Month 6 (N=260)**	
	**Positive n/N (%)**	**P-value**	**Positive n/N (%)**	**P-value**	**Positive n/N (%)**	**P-value**	**Positive n/N (%)**	**P-value**	**Positive n/N (%)**	**P-value**	**Positive n/N (%)**	**P-value**
**Overall**	58/301 (19.3)		57/276 (20.7)		48/273 (17.6)		4/270 (1.5)		3/255 (1.2)		16/260 (6.2)	
**Vaccination Site**		0.12		**<0.001**		**<0.001**		0.18		0.84		**0.003**
Adabraka PC	25/155 (16.1)		23/146 (15.8)		24/141 (17.0)		3/139 (2.2)		2/125 (1.6)		6/131 (4.6)	
Ridge Hospital	1/18 (5.6)		8/16 (50.0)		10/15 (66.7)		1/15 (6.7)		0/15 (0.0)		4/15 (26.7)	
Korle-Bu	10/39 (25.6)		2/38 (5.3)		1/38 (2.6)		0/38 (0.0)		0/37 (0.0)		0/37 (0.0)	
Legon Hospital	22/89 (24.7)		24/76 (31.6)		13/79 (16.5)		0/78 (0.0)		1/78 (1.3)		6/77 (7.8)	
**Age, median (IQR)**	29 (23-37)	0.32 [W]	30 (23-40)	0.26 [W]	31 (23-42)	0.046 [W]	27 (24-49)	0.68 [W]	23 (21-23)	0.095 [W]	35 (23-42)	0.31 [W]
**Age group (years)**		0.60		0.55		0.23		0.53		0.55		0.32
18-19	1/18 (5.6)		2/16 (12.5)		2/12 (16.7)		0/15 (0.0)		0/13 (0.0)		1/16 (6.3)	
20-29	33/157 (21.0)		26/141 (18.4)		18/139 (12.9)		3/135 (2.2)		3/127 (2.4)		6/129 (4.7)	
30-39	12/69 (17.4)		14/65 (21.5)		14/66 (21.2)		0/67 (0.0)		0/63 (0.0)		3/63 (4.8)	
40-49	6/29 (20.7)		7/28 (25.0)		6/29 (20.7)		0/27 (0.0)		0/26 (0.0)		4/26 (15.4)	
50+	6/28 (21.4)		8/26 (30.8)		8/27 (29.6)		1/26 (3.8)		0/26 (0.0)		2/26 (7.7)	
**Sex**		0.75		0.73		0.10		0.51		0.15		0.46
Female	24/119 (20.2)		22/112 (19.6)		24/108 (22.2)		1/111 (0.9)		0/102 (0.0)		8/107 (7.5)	
Male	34/182 (18.7)		35/164 (21.3)		24/165 (14.5)		3/159 (1.9)		3/153 (2.0)		8/153 (5.2)	
**Name of vaccine**		0.068		0.13		0.86		0.23		0.66		0.65
AstraZeneca	33/139 (23.7)		21/126 (16.7)		22/122 (18.0)		3/123 (2.4)		1/117 (0.9)		8/116 (6.9)	
Pfizer	25/162 (15.4)		36/150 (24.0)		26/151 (17.2)		1/147 (0.7)		2/138 (1.4)		8/144 (5.6)	
**Nationality**		0.33		0.37		<0.001		0.80		0.91		<0.001
Ghanaian	58/297 (19.5)		57/273 (20.9)		44/269 (16.4)		4/266 (1.5)		3/254 (1.2)		14/256 (5.5)	
Non-Ghanaian	0/4 (0.0)		0/3 (0.0)		4/4 (100.0)		0/4 (0.0)		0/1 (0.0)		2/4 (50.0)	
**Household size, Median (IQR)**	4 (2-6)	0.57 [W]	4 (2-5)	0.11 [W]	4 (2-6)	0.72 [W]	3 (2-4.5)	0.27 [W]	6 (3-7)	0.38 [W]	3 (1.5-5.5)	0.082 [W]
**Household size**		0.25		0.15		0.89		0.62		0.75		0.21
1-3 members	28/117 (23.9)		28/105 (26.7)		17/104 (16.3)		2/97 (2.1)		1/96 (1.0)		9/97 (9.3)	
4-6 members	21/134 (15.7)		21/128 (16.4)		22/123 (17.9)		2/126 (1.6)		1/115 (0.9)		6/116 (5.2)	
7+ members	9/50 (18.0)		8/43 (18.6)		9/46 (19.6)		0/47 (0.0)		1/44 (2.3)		1/47 (2.1)	
**Occupation-Category**		0.34		0.72		**0.001**		0.57		0.076		0.43
Formal	14/63 (22.2)		15/59 (25.4)		19/58 (32.8)		1/56 (1.8)		0/54 (0.0)		6/55 (10.9)	
Informal	27/120 (22.5)		20/109 (18.3)		20/113 (17.7)		1/108 (0.9)		0/107 (0.0)		5/106 (4.7)	
Student	15/97 (15.5)		19/90 (21.1)		9/82 (11.0)		1/86 (1.2)		3/78 (3.8)		4/81 (4.9)	
Unemployed	2/21 (9.5)		3/18 (16.7)		0/20 (0.0)		1/20 (5.0)		0/16 (0.0)		1/18 (5.6)	
**Average monthly income in GHC**		**0.011**		0.18		**0.016**		0.88		0.60		0.59
0-1000	27/190 (14.2)		32/175 (18.3)		21/173 (12.1)		3/171 (1.8)		3/158 (1.9)		10/162 (6.2)	
1001-2500	6/30 (20.0)		6/26 (23.1)		6/26 (23.1)		0/26 (0.0)		0/24 (0.0)		1/26 (3.8)	
Above 2500	6/15 (40.0)		6/14 (42.9)		5/15 (33.3)		0/13 (0.0)		0/15 (0.0)		2/14 (14.3)	
Prefer not to say	19/66 (28.8)		13/61 (21.3)		16/59 (27.1)		1/60 (1.7)		0/58 (0.0)		3/58 (5.2)	
**COVID-19 test history at baseline**		0.58		0.23		**0.019**		0.74		0.54		**<0.001**
Never tested	52/257 (20.2)		47/235 (20.0)		34/230 (14.8)		3/229 (1.3)		2/218 (0.9)		9/221 (4.1)	
Tested Negative	5/38 (13.2)		10/35 (28.6)		12/37 (32.4)		1/35 (2.9)		1/32 (3.1)		7/33 (21.2)	
Tested positive	1/6 (16.7)		0/6 (0.0)		2/6 (33.3)		0/6 (0.0)		0/5 (0.0)		0/6 (0.0)	
**Autoimmune disease**		0.28		**0.012**		**0.009**		0.41		0.47		0.093
No	48/262 (18.3)		55/238 (23.1)		47/235 (20.0)		4/232 (1.7)		3/218 (1.4)		16/223 (7.2)	
Yes	10/39 (25.6)		2/38 (5.3)		1/38 (2.6)		0/38 (0.0)		0/37 (0.0)		0/37 (0.0)	

**Co-morbid disease burden excluding autoimmune disease**		0.45			0.055			**0.030**			0.29			0.87			0.57
None	51/276 (18.5)			50/254 (19.7)			40/251 (15.9)			3/248 (1.2)			3/233 (1.3)			14/237 (5.9)	
1 condition	6/20 (30.0)			7/17 (41.2)			7/17 (41.2)			1/17 (5.9)			0/17 (0.0)			2/18 (11.1)	
2+ conditions	1/5 (20.0)			0/5 (0.0)			1/5 (20.0)			0/5 (0.0)			0/5 (0.0)			0/5 (0.0)	
**Number of COVID-19 symptoms at baseline**		**0.023**			**0.004**			0.12			0.89			**0.001**			**<0.001**
None	41/228 (18.0)			34/211 (16.1)			33/209 (15.8)			3/208 (1.4)			0/199 (0.0)			6/198 (3.0)	
1 symptom	11/61 (18.0)			20/56 (35.7)			11/54 (20.4)			1/52 (1.9)			3/48 (6.3)			7/52 (13.5)	
2+ symptoms	6/12 (50.0)			3/9 (33.3)			4/10 (40.0)			0/10 (0.0)			0/8 (0.0)			3/10 (30.0)	

W: Wilcoxon rank sum test of equality of median. All other tests are from the Pearson chi-square test.
IQR: interquartile range.

Table S3

Socio-demographics of participants included in the serological analysis,

Variables	Total N (%)	Anti-N status @ baseline: Anti-N Negative (aNN) N (%)	Anti-N positive (ANP) N (%)	P-value
**Total**	**231**	**45**	**186**	**No test**
**Age**				0.1660 W
Min / Max	18.0 / 78.0	19.0 / 67.0	18.0 / 78.0	
Med [IQR]	28.0 [23.0;37.0]	31.0 [23.0;39.0]	27.0 [23.0;36.8]	
Mean (std)	31.4 (11.4)	33.2 (11.7)	31.0 (11.3)	
**Age group**				0.5633 F
18-19	13 (5.6%)	1 (2.2%)	12 (6.5%)	
20-29	115 (49.8%)	20 (44.4%)	95 (51.1%)	
30-39	57 (24.7%)	14 (31.1%)	43 (23.1%)	
40-49	23 (10.0%)	4 (8.9%)	19 (10.2%)	
50+	23 (10.0%)	6 (13.3%)	17 (9.1%)	
**Sex**				0.0093 C
Female	94 (40.7%)	26 (57.8%)	68 (36.6%)	
Male	137 (59.3%)	19 (42.2%)	118 (63.4%)	
**Occupation category**				0.0016 F
Formal	47 (20.3%)	19 (42.2%)	28 (15.1%)	
Informal	100 (43.3%)	14 (31.1%)	86 (46.2%)	
Student	70 (30.3%)	11 (24.4%)	59 (31.7%)	
Unemployed	14 (6.1%)	1 (2.2%)	13 (7.0%)	
**Average monthly income in GHC**				0.0063 F
0-1000	147 (63.6%)	28 (62.2%)	119 (64.0%)	
1001-2500	21 (9.1%)	8 (17.8%)	13 (7.0%)	
Above 2500	13 (5.6%)	5 (11.1%)	8 (4.3%)	
Prefer not to say	50 (21.6%)	4 (8.9%)	46 (24.7%)	
**Household size**				0.3031 F
1 member	17 (7.4%)	2 (4.4%)	15 (8.1%)	
2-3 members	72 (31.2%)	13 (28.9%)	59 (31.7%)	
4-5 members	76 (32.9%)	12 (26.7%)	64 (34.4%)	
≥6 members	66 (28.6%)	18 (40.0%)	48 (25.8%)	
**COVID-19 test history at baseline**				0.0154 F
Negative	27 (11.7%)	11 (24.4%)	16 (8.6%)	
Positive	5 (2.2%)	1 (2.2%)	4 (2.2%)	
Never tested	199 (86.1%)	33 (73.3%)	166 (89.2%)	
**Covid-19 test @ baseline**				0.1127 C
Negative	184 (79.7%)	32 (71.1%)	152 (81.7%)	
Positive	47 (20.3%)	13 (28.9%)	34 (18.3%)	
**Do you have any underlying conditions?**				0.0425 C
No	180 (77.9%)	30 (66.7%)	150 (80.6%)	
Yes	51 (22.1%)	15 (33.3%)	36 (19.4%)	
**Asthma**				0.0978 F
No	228 (98.7%)	43 (95.6%)	185 (99.5%)	
Yes	3 (1.3%)	2 (4.4%)	1 (0.5%)	
**Autoimmune disease**				0.3628 C
No	195 (84.4%)	36 (80.0%)	159 (85.5%)	
Yes	36 (15.6%)	9 (20.0%)	27 (14.5%)	
**Diabetes**				0.0978 F
No	228 (98.7%)	43 (95.6%)	185 (99.5%)	
Yes	3 (1.3%)	2 (4.4%)	1 (0.5%)	
**Fatty liver**				1.0000 F
No	230 (99.6%)	45 (100.0%)	185 (99.5%)	
Yes	1 (0.4%)	0 (0%)	1 (0.5%)	
**Heart disease/hypertension**				0.7215 F
No	218 (94.4%)	42 (93.3%)	176 (94.6%)	
Yes	13 (5.6%)	3 (6.7%)	10 (5.4%)	
**Kidney disease**				1.0000 F
No	229 (99.1%)	45 (100.0%)	184 (98.9%)	
Yes	2 (0.9%)	0 (0%)	2 (1.1%)	
**Sinusitis**				1.0000 F
No	230 (99.6%)	45 (100.0%)	185 (99.5%)	
Yes	1 (0.4%)	0 (0%)	1 (0.5%)	
**Stomach ulcer**				1.0000 F
No	230 (99.6%)	45 (100.0%)	185 (99.5%)	
Yes	1 (0.4%)	0 (0%)	1 (0.5%)	
**Disease burden excluding auto-immune disease**				0.2324 F
None	212 (91.8%)	39 (86.7%)	173 (93.0%)	
1 condition	14 (6.1%)	5 (11.1%)	9 (4.8%)	
≥2 conditions	5 (2.2%)	1 (2.2%)	4 (2.2%)	

**Are you on any medication for the above conditions?**				0.3835 [F]
No	7 (14.0%)	3 (21.4%)	4 (11.1%)	
Yes	43 (86.0%)	11 (78.6%)	32 (88.9%)	
NA	181	31	150	
**Had COVID-19 symptoms at baseline**				0.4818 [C]
No	149 (64.5%)	27 (60.0%)	122 (65.6%)	
Yes	82 (35.5%)	18 (40.0%)	64 (34.4%)	
**Body pain**				0.3121 [C]
No	196 (84.8%)	36 (80.0%)	160 (86.0%)	
Yes	35 (15.2%)	9 (20.0%)	26 (14.0%)	
**Cough**				0.3317 [F]
No	225 (97.4%)	43 (95.6%)	182 (97.8%)	
Yes	6 (2.6%)	2 (4.4%)	4 (2.2%)	
**Dizziness**				1.0000 [F]
No	230 (99.6%)	45 (100.0%)	185 (99.5%)	
Yes	1 (0.4%)	0 (0%)	1 (0.5%)	
**Fatigue**				1.0000 [F]
No	229 (99.1%)	45 (100.0%)	184 (98.9%)	
Yes	2 (0.9%)	0 (0%)	2 (1.1%)	
**Fever**				0.0749 [F]
No	222 (96.1%)	41 (91.1%)	181 (97.3%)	
Yes	9 (3.9%)	4 (8.9%)	5 (2.7%)	
**Headache**				0.5530 [C]
No	199 (86.1%)	40 (88.9%)	159 (85.5%)	
Yes	32 (13.9%)	5 (11.1%)	27 (14.5%)	
**Los of smell taste**				1.0000 [F]
No	230 (99.6%)	45 (100.0%)	185 (99.5%)	
Yes	1 (0.4%)	0 (0%)	1 (0.5%)	
**Sore throat**				1.0000 [F]
No	228 (98.7%)	45 (100.0%)	183 (98.4%)	
Yes	3 (1.3%)	0 (0%)	3 (1.6%)	
**Cold**				0.1948 [F]
No	230 (99.6%)	44 (97.8%)	186 (100.0%)	
Yes	1 (0.4%)	1 (2.2%)	0 (0%)	
**Stomachache**				1.0000 [F]
No	230 (99.6%)	45 (100.0%)	185 (99.5%)	
Yes	1 (0.4%)	0 (0%)	1 (0.5%)	
**Number of COVID-19 symptoms at baseline**				0.3456 [F]
None	149 (64.5%)	27 (60.0%)	122 (65.6%)	
1 symptom	74 (32.0%)	15 (33.3%)	59 (31.7%)	
≥2 symptoms	8 (3.5%)	3 (6.7%)	5 (2.7%)	

Table S4
Anti-N and anti-S positivity and values during the HERITAGE study period.

Variable	Timepoint Baseline	Month 3	Month 6	Month 12	Total	P-value
Total	231 (25.0%)	231 (25.0%)	231 (25.0%)	231 (25.0%)	921 (100.0%)	
Anti-N status						
Negative	45 (19.5%)	17 (7.4%)	12 (5.2%)	7 (3.0%)	81 (8.8%)	
Positive	186 (80.5%)	213 (92.6%)	218 (94.8%)	223 (97.0%)	840 (91.2%)	
NA	0	1	1	1		<0.0001
Anti-N positives values						
Min / Max	1.1 / 251.3	1.3 / 246.4	1.7 / 250.7	1.2 / 250.0	1.1 / 251.3	
Med [IQR]	79.5 [9.7;147.1]	63.6 [18.9;170.0]	76.3 [18.9;168.0]	73.8 [29.2;167.9]	73.7 [20.5;162.1]	
Mean (std)	85.1 (72.8)	92.9 (79.8)	94.6 (76.0)	97.5 (76.2)	92.8 (76.3)	0.1587
Anti-S status						
Negative	41 (17.7%)	1 (0.4%)	1 (0.4%)	2 (0.9%)	45 (4.9%)	
Positive	190 (82.3%)	229 (99.6%)	229 (99.6%)	228 (99.1%)	876 (95.1%)	
NA	0	1	1	1		<0.0001
Anti-S positives values						
Min / Max	2.1 / 25,000	42.4 / 25,000	1.8 / 25,000	138.2 / 25,000	1.8 / 25,000	
Med [IQR]	1284.5 [183.0;5470.2]	5548.0 [1859.0;10,660.2]	4047.0 [2027.0;7008.0]	3473.5 [1786.8;6533.2]	3841.5 [1396.8;7345.2]	
Mean (std)	3669.1 (5051.5)	7531.9 (6984.4)	5570.7 (5146.4)	4630.2 (3973.8)	5426.2 (5587.7)	<0.0001

Table S5
Neutralising antibody titres during the HERITAGE study period

	Timepoint					
**Variables**	**Baseline**	**Month 3**	**Month 6**	**Month 12**	**Total**	**P-value**
**Wild-type variant**						
Min / Max	5.0 / 5120.0	57.3 / 5120.0	83.2 / 5120.0	53.4 / 5120.0	5.0 / 5120.0	
Med [IQR]	307.5 [159.6;808.5]	943.0 [571.5;1945.2]	795.9 [486.7;1329.0]	726.5 [428.5;1084.5]	726.5 [338.3;1198.4]	<0.0001K
Mean (std)	718.8 (1120.6)	1785.8 (1821.6)	1356.2 (1510.0)	1096.6 (1282.5)	1238.5 (1505.8)	
**Wild-type category**						<0.0001F
<40	1 (0.4%)	0 (0%)	0 (0%)	0 (0%)	1 (0.1%)	
40-256	101 (43.7%)	21 (9.1%)	18 (7.9%)	33 (14.3%)	173 (18.8%)	
>256	129 (55.8%)	209 (90.9%)	211 (92.1%)	198 (85.7%)	747 (81.1%)	
NA	0	1	2	0	3	
**A.27 variant**						
Min / Max	5.0 / 5120.0	5.0 / 5120.0	5.0 / 5120.0	5.0 / 5120.0	5.0 / 5120.0	
Med [IQR]	134.2 [41.8;633.2]	616.2 [204.5;1228.7]	511.9 [214.7;921.1]	526.2 [187.5;967.3]	474.1 [158.9;908.1]	<0.0001K
Mean (std)	591.0 (1189.6)	1189.0 (1569.4)	976.7 (1338.2)	876.7 (1168.8)	908.3 (1341.4)	
**A.27 category**						<0.0001#
<40	54 (23.4%)	3 (1.3%)	5 (2.2%)	4 (1.7%)	66 (7.2%)	
40-256	90 (39.0%)	61 (26.4%)	56 (24.3%)	63 (27.3%)	270 (29.3%)	
>256	87 (37.7%)	167 (72.3%)	169 (73.5%)	164 (71.0%)	587 (63.6%)	
NA	0	0	1	0	1	
**Eta variant**						
Min / Max	5.0 / 5120.0	43.5 / 5120.0	47.6 / 5120.0	5.0 / 5120.0	5.0 / 5120.0	
Med [IQR]	206.7 [82.1;589.7]	720.8 [362.9;1352.0]	555.9 [354.9;933.9]	519.4 [325.4;790.3]	505.0 [244.7;942.1]	<0.0001K
Mean (std)	516.9 (914.5)	1184.5 (1359.1)	779.1 (868.6)	651.1 (591.1)	782.9 (1003.2)	
**Eta category**						<0.0001#
<40	21 (9.1%)	0 (0%)	0 (0%)	2 (0.9%)	23 (2.5%)	
40-256	115 (49.8%)	39 (16.9%)	35 (15.2%)	38 (16.5%)	227 (24.6%)	
>256	95 (41.1%)	192 (83.1%)	195 (84.8%)	191 (82.7%)	673 (72.9%)	
NA	0	0	1	0	1	
**Delta variant**						
Min / Max	5.0 / 5120.0	5.0 / 5120.0	5.0 / 5120.0	5.0 / 5120.0	5.0 / 5120.0	
Med [IQR]	189.2 [85.9;509.9]	562.7 [291.9;989.1]	478.1 [298.5;807.5]	426.8 [297.9;691.7]	426.4 [205.6;772.3]	<0.0001K
Mean (std)	519.5 (976.6)	961.0 (1220.1)	740.9 (939.3)	561.1 (496.7)	695.3 (959.0)	
**Delta category**						<0.0001 #
<40	25 (10.8%)	3 (1.3%)	4 (1.7%)	2 (0.9%)	34 (3.7%)	
40-256	115 (49.8%)	43 (18.7%)	44 (19.1%)	42 (18.2%)	244 (26.5%)	
>256	91 (39.4%)	184 (80.0%)	182 (79.1%)	187 (81.0%)	644 (69.8%)	
NA	0	1	1	0	2	
**Omicron BA.2 variant**						
Min / Max	5.0 / 5120.0	5.0 / 5120.0	5.0 / 5120.0	5.0 / 5120.0	5.0 / 5120.0	
Med [IQR]	175.0 [86.1;397.9]	330.7 [178.2;521.4]	364.3 [189.9;565.4]	366.7 [227.0;528.3]	323.8 [173.9;520.0]	<0.0001K
Mean (std)	297.5 (428.0)	435.3 (590.7)	535.8 (761.7)	461.9 (456.8)	432.5 (580.0)	
**Omicron BA.2 category**						<0.0001#
<40	20 (8.7%)	3 (1.3%)	3 (1.3%)	2 (0.9%)	28 (3.0%)	
40-256	128 (55.4%)	93 (40.3%)	75 (32.6%)	63 (27.3%)	359 (38.9%)	
>256	83 (35.9%)	135 (58.4%)	152 (66.1%)	166 (71.9%)	536 (58.1%)	
NA	0	0	1	0	1	
**XBB.1.5 variant**						
Min / Max	5.0 / 2513.4	5.0 / 749.2	5.0 / 825.0	5.0 / 1325.5	5.0 / 2513.4	<0.0001K
Med [IQR]	65.7 [10.0;117.6]	126.5 [73.5;183.7]	101.6 [62.9;165.5]	134.3 [70.6;178.1]	101.8 [54.9;169.9]	<0.0001K
Mean (std)	97.3 (185.7)	145.5 (110.2)	129.2 (117.5)	156.0 (155.5)	132.0 (146.8)	<0.0001K
**XBB.1.5 category**						<0.0001#
<40	63 (27.4%)	20 (8.7%)	33 (14.3%)	20 (8.7%)	136 (14.8%)	
40-256	156 (67.8%)	189 (81.8%)	174 (75.7%)	184 (79.7%)	703 (76.2%)	
>256	11 (4.8%)	22 (9.5%)	23 (10.0%)	27 (11.7%)	83 (9.0%)	
NA	1	0	1	0	2	

K: P-value from the Kruskal Wallis test. F: P-value from the Fisher's exact test. #: P-value from the Pearson chi-square test.

Table S6: Comparison of neutralisation level of Heritage aNN, and Heritage aNP samples at the different timepoints.

	**Baseline neutralization level**			**Month 3 neutralization level**		
**Variable**	**Heritage aNN**	**Heritage aNP**		**Heritage aNN**	**Heritage aNP**	
**N**	**45**	**186**	**P-value**	**45**	**186**	**P-value**
**Wildtype**			<0.0001			<0.0001
Min / Max	5.0 / 548.8	66.8 / 5120.0		57.3 / 5120.0	156.9 / 5120.0	
Med [IQR]	143.7 [101.8;177.8]	437.2 [202.5;919.9]		413.9 [179.8;745.9]	1104.6 [732.3;2524.4]	
Mean (std)	153.6 (85.1)	855.5 (1209.6)		867.6 (1389.1)	2009.2 (1847.2)	
**A. 27**			<0.0001			<0.0001
Min / Max	5.0 / 108.9	5.0 / 5120.0		5.0 / 5120.0	47.7 / 5120.0	
Med [IQR]	5.0 [5.0;5.0]	191.5 [57.5;784.1]		146.1 [49.2;296.4]	729.1 [421.1;1338.4]	
Mean (std)	14.1 (22.3)	730.6 (1287.9)		480.9 (1096.6)	1360.3 (1620.0)	
**Eta**			<0.0001			<0.0001
Min / Max	5.0 / 196.1	5.0 / 5120.0		43.5 / 5120.0	104.1 / 5120.0	
Med [IQR]	45.7 [10.0;63.9]	279.2 [133.1;723.8]		218.3 [113.3;367.0]	874.9 [518.4;1483.2]	
Mean (std)	48.3 (46.6)	630.3 (986.4)		565.5 (1080.9)	1334.2 (1379.4)	
**Delta**			<0.0001			<0.0001
Min / Max	5.0 / 238.7	5.0 / 5120.0		5.0 / 5120.0	76.4 / 5120.0	
Med [IQR]	42.8 [5.0;70.3]	248.6 [133.1;616.4]		196.8 [99.3;319.9]	646.6 [413.5;1166.3]	
Mean (std)	49.4 (50.5)	633.3 (1057.5)		594.3 (1260.0)	1050.2 (1196.7)	
**Omicron BA.2**			<0.0001			<0.0001
Min / Max	5.0 / 257.4	5.0 / 5120.0		5.0 / 1098.8	5.0 / 5120.0	
Med [IQR]	63.1 [10.0;87.3]	200.6 [135.5;482.1]		164.6 [133.8;231.3]	386.9 [210.2;580.5]	
Mean (std)	67.3 (55.5)	353.2 (459.4)		227.8 (214.8)	485.5 (640.2)	
**XBB 1.5**			<0.0001			0.0008
Min / Max	5.0 / 109.9	5.0 / 2513.4		5.0 / 705.8	5.0 / 749.2	
Med [IQR]	10.0 [5.0;50.7]	74.9 [44.3;139.3]		83.7 [42.2;142.4]	144.5 [85.2;188.3]	
Mean (std)	31.0 (29.2)	113.4 (203.4)		115.1 (124.3)	152.9 (105.6)	

	**Month 6 neutralization level**			**Month 12 neutralization level**		
**Variable**	**Heritage aNN**	**Heritage aNP**		**Heritage aNN**	**Heritage aNP**	
**N**	**45**	**186**	**P-value**	**45**	**186**	**P-value**
**Wildtype**			<0.0001			0.0003
Min / Max	90.3 / 5120.0	83.2 / 5120.0		53.4 / 5120.0	78.3 / 5120.0	
Med [IQR]	340.7 [223.5;731.0]	872.2 [593.0;1381.6]		383.8 [196.1;940.9]	780.0 [544.9;1109.7]	
Mean (std)	1029.6 (1647.6)	1436.1 (1468.1)		918.5 (1395.3)	1139.7 (1253.9)	
**A. 27**			<0.0001			<0.0001
Min / Max	5.0 / 5120.0	5.0 / 5120.0		5.0 / 5120.0	10.0 / 5120.0	
Med [IQR]	162.6 [46.6;566.4]	574.9 [378.2;976.2]		178.9 [116.4;474.9]	576.2 [312.7;1025.1]	
Mean (std)	522.2 (899.8)	1087.2 (1404.4)		595.3 (1106.1)	944.8 (1176.2)	
**Eta**			<0.0001			<0.0001
Min / Max	47.6 / 2098.2	91.3 / 5120.0		10.0 / 5120.0	5.0 / 5120.0	
Med [IQR]	235.6 [135.0;464.2]	634.8 [401.4;967.7]		304.5 [206.9;529.5]	548.4 [370.4;858.6]	
Mean (std)	451.3 (471.8)	858.8 (923.6)		532.3 (808.6)	679.8 (524.1)	
**Delta**			<0.0001			0.0211
Min / Max	5.0 / 5120.0	10.0 / 5120.0		5.0 / 2422.6	43.6 / 5120.0	
Med [IQR]	218.8 [139.6;575.9]	516.2 [340.4;841.1]		333.6 [228.8;584.1]	437.2 [320.4;694.2]	
Mean (std)	685.1 (1268.6)	754.4 (843.9)		488.5 (453.8)	578.7 (506.1)	
**Omicron BA.2**			0.0060			0.9208
Min / Max	5.0 / 5120.0	10.0 / 5120.0		5.0 / 5120.0	5.0 / 2399.3	
Med [IQR]	205.3 [160.5;528.5]	387.9 [225.5;566.4]		402.1 [197.7;640.4]	362.4 [239.8;520.2]	
Mean (std)	593.0 (1078.4)	521.8 (665.6)		580.9 (798.2)	433.1 (321.9)	
**XBB 1.5**			0.2175			0.2941
Min / Max	5.0 / 739.4	5.0 / 825.0		5.0 / 653.4	5.0 / 1325.5	
Med [IQR]	80.7 [50.0;169.1]	103.5 [68.4;164.2]		159.5 [71.1;228.6]	130.7 [70.0;173.3]	
Mean (std)	134.4 (152.9)	127.9 (107.6)		171.6 (146.0)	152.3 (157.9)	

Table S7
Demographics for matched HERITAGE and LEGACY participants

Characteristics	HERITAGE matched to LEGACY<br>N (%)	LEGACY matched to HERITAGE<br>N (%)
**Overall**	159 (100.0)	312 (100.0)
**Vaccination Site**		
Adabraka Polyclinic	82 (51.6)	-
Ridge Hospital	8 (5.0)	-
Korle-Bu	36 (22.6)	-
Legon Hospital	33 (20.8)	-
**Age, Median (IQR)**	33 (26-40)	34 (29-43)
**Age group (years)**		
18-19	5 (3.1)	0 (0)
20-29	53 (33.3)	86 (27.6)
30-39	57 (35.8)	116 (37.2)
40-49	22 (13.8)	70 (22.4)
50+	22 (13.8)	40 (12.8)
**Sex**		
Female	93 (58.5)	201 (64.4)
Male	66 (41.5)	111 (35.6)
**Name of vaccine**		
AstraZeneca	84 (52.8)	140 (44.9)
Pfizer	75 (47.2)	172 (55.1)
**Nationality**		
Ghanaian	159 (100.0)	-
Non-Ghanaian	-	-
**Household size, Median (IQR)**	4 (3-6)	-
**Household size**		
1-3 members	58 (36.5)	-
4-6 members	75 (47.2)	-
7+ members	26 (16.4)	-
**Occupation-Category**		
Formal	40 (25.2)	-
Informal	76 (47.8)	-
Student	37 (23.3)	-
Unemployed	6 (3.8)	-
**Average monthly income in GHC**		
0-1000	98 (61.6)	-
1001-2500	19 (11.9)	-
Above 2500	8 (5.0)	-
Prefer not to say	34 (21.4)	-
**COVID-19 test history at baseline**		
Never tested	132 (83.0)	-
Tested Negative	22 (13.8)	48 (15.4)
Tested positive	5 (3.1)	264 (84.6)
**Autoimmune disease**		
No	123 (77.4)	-
Yes	36 (22.6)	-
**Co-morbid disease burden excluding autoimmune disease**		
None	141 (88.7)	-
1 condition	13 (8.2)	-
2+ conditions	5 (3.1)	-
**Number of COVID-19 symptoms at baseline**		
None	96 (60.4)	-
1 symptom	56 (35.2)	-
2+ symptoms	7 (4.4)	-

Table S8

Comparison of neutralisation levels of matched samples between HERITAGE and LEGACY

	Baseline			Month 3			Month 6			Month 12		
**Variants & statistics**	**Heritage**	**Legacy**	**P-value**	**Heritage**	**Legacy**	**P-value**	**Heritage**	**Legacy**	**P-value**	**Heritage**	**Legacy**	**P-value**
**Wildtype**												
Min / Max	5.0 / 5120.0	5.0 / 5120.0		57.3 / 5120.0	5.0 / 5120.0		83.2 / 5120.0	10.0 / 5120.0		53.4 / 5120.0	10.0 / 5120.0	
Med [IQR]	250.3 [152.8;701.4]	141.3 [111.7;222.8]	<0.0001	882.6 [517.5;2047.4]	337.7 [171.1;983.5]	<0.0001	810.7 [467.5;1308.2]	228.5 [143.9;598.1]	<0.0001	715.2 [407.8;1128.1]	325.5 [162.4;958.9]	0.0002
Mean (std)	661.0 (1099.1)	406.5 (944.4)		1788.3 (1889.2)	1126.8 (1715.3)		1381.2 (1565.2)	869.8 (1539.5)		1219.4 (1455.0)	1053.3 (1597.3)	
N (NA)	142 (0)	142 (0)		155 (1)	156 (0)		153 (1)	154 (0)		109 (0)	108 (1)	
**A27**												
Min / Max	5.0 / 5120.0	5.0 / 5120.0		5.0 / 5120.0	5.0 / 5120.0		5.0 / 5120.0	5.0 / 5120.0		5.0 / 5120.0	5.0 / 5120.0	
Med [IQR]	84.6 [10.0;559.8]	43.2 [10.0;95.9]	0.0129	552.0 [199.6;1248.9]	227.7 [101.8;656.9]	0.0001	496.4 [198.3;905.0]	180.5 [59.8;770.5]	0.0003	474.9 [174.6;1008.4]	339.3 [51.8;1754.9]	0.3252
Mean (std)	566.7 (1196.0)	582.7 (1428.6)		1189.7 (1625.7)	951.5 (1644.1)		939.1 (1318.9)	968.5 (1668.9)		930.4 (1303.4)	1409.8 (1973.2)	
N (NA)	142 (0)	141 (1)		156 (0)	156 (0)		154 (0)	153 (1)		109 (0)	106 (3)	
**Eta**												
Min / Max	5.0 / 5120.0	10.0 / 5120.0		43.5 / 5120.0	55.9 / 5120.0		55.2 / 5120.0	43.4 / 5120.0		5.0 / 5120.0	40.1 / 5120.0	
Med [IQR]	159.7 [67.8;552.4]	118.1 [78.2;206.5]	0.3428	628.2 [315.7;1260.1]	295.3 [173.9;625.5]	<0.0001	548.6 [340.9;933.9]	272.7 [152.2;566.4]	<0.0001	518.0 [315.1;884.9]	319.5 [138.7;883.8]	0.0249
Mean (std)	459.3 (878.5)	521.6 (1193.0)		1201.1 (1472.8)	582.1 (863.9)		753.5 (829.9)	750.7 (1325.1)		678.9 (642.8)	944.6 (1456.8)	
N (NA)	142 (0)	141 (1)		156 (0)	156 (0)		154 (0)	153 (1)		109 (0)	106 (3)	
**Delta**												
Min / Max	5.0 / 5120.0	5.0 / 5120.0		5.0 / 5120.0	5.0 / 5120.0		10.0 / 5120.0	5.0 / 5120.0		5.0 / 2422.6	5.0 / 5120.0	
Med [IQR]	158.6 [56.1;461.2]	10.0 [5.0;91.5]	<0.0001	557.6 [288.8;1162.7]	165.1 [65.8;337.3]	<0.0001	482.3 [278.4;886.9]	161.8 [61.6;376.9]	<0.0001	430.9 [297.2;722.7]	209.8 [71.1;943.7]	0.0018
Mean (std)	546.3 (1128.0)	293.6 (959.4)		1020.6 (1312.9)	312.9 (527.0)		785.2 (997.4)	682.6 (1440.4)		558.2 (412.2)	865.6 (1465.7)	
N (NA)	142 (0)	142 (0)		155 (1)	156 (0)		154 (0)	154 (0)		109 (0)	108 (1)	
**Omicron BA2**												
Min / Max	5.0 / 5120.0	5.0 / 5120.0		5.0 / 5120.0	5.0 / 860.4		10.0 / 5120.0	5.0 / 5120.0		5.0 / 2006.5	5.0 / 5120.0	
Med [IQR]	153.7 [76.7;350.3]	10.0 [5.0;86.8]	<0.0001	283.3 [173.9;496.5]	124.2 [70.5;223.5]	<0.0001	357.8 [182.1;571.3]	131.5 [80.7;295.1]	<0.0001	402.1 [269.1;560.0]	157.0 [70.1;497.3]	<0.0001
Mean (std)	291.1 (508.5)	221.9 (695.3)		457.4 (705.9)	195.3 (198.4)		539.8 (815.3)	319.3 (589.6)		448.7 (308.1)	563.7 (1103.7)	
N (NA)	142 (0)	137 (5)		156 (0)	69 (87)		154 (0)	99 (55)		109 (0)	104 (5)	

IQR: interquartile range. Std: Standard deviation. N(NA): Number of valid cases (Number of missing observation)

Table S9

Comparison of neutralisation levels of matched samples between the aNN subgroup in HERITAGE and LEGACY

Variable	Baseline neutralisation level			Month 3 neutralisation level		
	Heritage aNN	Legacy		Heritage aNN	Legacy	
**N**	**36**	**142**	**P-value**	**36**	**156**	**P-value**
**Wildtype**			0.1127			0.9721
Min / Max	5.0 / 548.8	5.0 / 5120.0		57.3 / 5120.0	5.0 / 5120.0	
Med [IQR]	136.6 [101.4;169.7]	141.3 [111.7;222.8]		347.9 [185.6;569.4]	337.7 [171.1;983.5]	
Mean (std)	149.9 (85.0)	406.5 (944.4)		907.0 (1534.0)	1126.8 (1715.3)	
**A. 27**			<0.0001			0.0070
Min / Max	5.0 / 108.9	5.0 / 5120.0		5.0 / 5120.0	5.0 / 5120.0	
Med [IQR]	5.0 [5.0;5.0]	43.2 [10.0;95.9]		137.0 [49.6;264.7]	227.7 [101.8;656.9]	
Mean (std)	14.2 (22.5)	582.7 (1428.6)		519.7 (1215.9)	951.5 (1644.1)	
**Eta**			<0.0001			0.0070
Min / Max	5.0 / 196.1	10.0 / 5120.0		43.5 / 5120.0	55.9 / 5120.0	
Med [IQR]	45.7 [10.0;63.6]	118.1 [78.2;206.5]		193.4 [124.2;315.7]	295.3 [173.9;625.5]	
Mean (std)	47.6 (46.7)	521.6 (1193.0)		578.3 (1190.5)	582.1 (863.9)	
**Delta**			0.9076			0.2816
Min / Max	5.0 / 238.7	5.0 / 5120.0		5.0 / 5120.0	5.0 / 5120.0	
Med [IQR]	41.4 [5.0;66.9]	10.0 [5.0;91.5]		190.8 [127.0;298.6]	165.1 [65.8;337.3]	
Mean (std)	45.9 (52.6)	293.6 (959.4)		648.9 (1398.4)	312.9 (527.0)	
**Omicron BA.2**			0.0304			0.1357
Min / Max	5.0 / 257.4	5.0 / 5120.0		5.0 / 1098.8	5.0 / 860.4	
Med [IQR]	61.1 [10.0;81.6]	10.0 [5.0;86.8]		161.9 [121.3;200.2]	124.2 [70.5;223.5]	
Mean (std)	64.6 (53.3)	221.9 (695.3)		223.7 (225.4)	195.3 (198.4)	

Variable	Month 6 neutralisation level			Month 12 neutralisation level		
	Heritage aNN	Legacy		Heritage aNN	Legacy	
**N**	**35**	**154**	**P-value**	**36**	**108**	**P-value**
**Wildtype**			0.0458			0.4793
Min / Max	107.3 / 5120.0	10.0 / 5120.0		53.4 / 5120.0	10.0 / 5120.0	
Med [IQR]	326.7 [210.6;850.3]	228.5 [143.9;598.1]		407.8 [194.5;1066.9]	325.5 [162.4;958.9]	
Mean (std)	1205.4 (1833.0)	869.8 (1539.5)		1076.4 (1537.6)	1053.3 (1597.3)	
**A. 27**			0.3730			0.5025
Min / Max	5.0 / 5120.0	5.0 / 5120.0		5.0 / 5120.0	5.0 / 5120.0	
Med [IQR]	150.1 [46.5;817.6]	180.5 [59.8;770.5]		178.9 [109.5;851.8]	339.3 [51.8;1754.9]	
Mean (std)	603.1 (1003.4)	968.5 (1668.9)		786.2 (1367.6)	1409.8 (1973.2)	
**Eta**			0.5852			0.8755
Min / Max	55.2 / 2098.2	43.4 / 5120.0		10.0 / 5120.0	40.1 / 5120.0	
Med [IQR]	196.5 [133.8;860.4]	272.7 [152.2;566.4]		268.2 [186.7;632.4]	319.5 [138.7;883.8]	
Mean (std)	488.3 (524.5)	750.7 (1325.1)		629.5 (1012.6)	944.6 (1456.8)	
**Delta**			0.0571			0.2282
Min / Max	10.0 / 5120.0	5.0 / 5120.0		5.0 / 2422.6	5.0 / 5120.0	
Med [IQR]	213.2 [133.4;694.3]	161.8 [61.6;376.9]		337.4 [234.8;814.7]	209.8 [71.1;943.7]	
Mean (std)	794.4 (1418.3)	682.6 (1440.4)		553.3 (533.4)	865.6 (1465.7)	
**Omicron BA.2**			0.0294			0.0162
Min / Max	10.0 / 5120.0	5.0 / 5120.0		5.0 / 1527.7	5.0 / 5120.0	
Med [IQR]	189.9 [148.1;551.8]	131.5 [80.7;295.1]		470.0 [235.7;648.6]	157.0 [70.1;497.3]	
Mean (std)	620.5 (1194.3)	319.3 (589.6)		517.6 (413.1)	563.7 (1103.7)	

aNN: anti-N negative at baseline.

Table S10

Comparison of neutralisation level of matched samples between Heritage aNN, and Heritage aNP

	**Baseline neutralisation level**			**Month 3 neutralisation level**		
**Variable**	**Heritage aNN**	**Heritage aNP**		**Heritage aNN**	**Heritage aNP**	
**N**	**36**	**106**	**P-value**	36	105	**P-value**
**Wildtype**			<0.0001			<0.0001
Min / Max	5.0 / 548.8	66.8 / 5120.0		57.3 / 5120.0	167.7 / 5120.0	
Med [IQR]	136.6 [101.4;169.7]	434.3 [200.0;829.5]		347.9 [185.6;569.4]	1057.6 [732.8;5120.0]	
Mean (std)	149.9 (85.0)	834.6 (1224.7)		907.0 (1534.0)	2147.0 (1945.3)	
**A.27**			<0.0001			<0.0001
Min / Max	5.0 / 108.9	5.0 / 5120.0		5.0 / 5120.0	47.7 / 5120.0	
Med [IQR]	5.0 [5.0;5.0]	191.5 [51.9;803.0]		137.0 [49.6;264.7]	710.2 [461.4;1416.8]	
Mean (std)	14.2 (22.5)	754.4 (1334.3)		519.7 (1215.9)	1475.0 (1725.6)	
**Eta**			<0.0001			<0.0001
Min / Max	5.0 / 196.1	5.0 / 5120.0		43.5 / 5120.0	126.4 / 5120.0	
Med [IQR]	45.7 [10.0;63.6]	255.5 [119.9;710.6]		193.4 [124.2;315.7]	880.3 [521.2;1556.1]	
Mean (std)	47.6 (46.7)	599.1 (978.6)		578.3 (1190.5)	1459.8 (1531.5)	
**Delta**			<0.0001			<0.0001
Min / Max	5.0 / 238.7	5.0 / 5120.0		5.0 / 5120.0	76.4 / 5120.0	
Med [IQR]	41.4 [5.0;66.9]	223.2 [94.3;552.1]		190.8 [127.0;298.6]	690.2 [428.7;1390.9]	
Mean (std)	45.9 (52.6)	716.3 (1261.9)		648.9 (1398.4)	1174.6 (1277.4)	
**Omicron BA.2**			<0.0001			<0.0001
Min / Max	5.0 / 257.4	5.0 / 5120.0		5.0 / 1098.8	5.0 / 5120.0	
Med [IQR]	61.1 [10.0;81.6]	190.3 [130.6;464.2]		161.9 [121.3;200.2]	398.2 [189.4;594.9]	
Mean (std)	64.6 (53.3)	368.0 (568.0)		223.7 (225.4)	553.7 (831.4)	
**XBB.1.5**			<0.0001			0.0002
Min / Max	5.0 / 109.9	5.0 / 2513.4		5.0 / 425.3	5.0 / 749.2	
Med [IQR]	10.0 [8.8;54.5]	73.9 [45.5;124.6]		82.7 [44.7;135.3]	157.0 [95.6;211.1]	
Mean (std)	32.6 (29.6)	128.0 (260.4)		104.1 (90.7)	169.4 (114.4)	

	**Month 6 neutralisation level**			**Month 12 neutralisation level**		
**Variable**	**Heritage aNN**	**Heritage aNP**		**Heritage aNN**	**Heritage aNP**	
**N**	**35**	**104**	**P-value**	**36**	**106**	**P-value**
**Wildtype**			<0.0001			0.0506
Min / Max	107.3 / 5120.0	83.2 / 5120.0		53.4 / 5120.0	78.3 / 5120.0	
Med [IQR]	326.7 [210.6;850.3]	872.2 [579.1;1403.7]		407.8 [194.5;1066.9]	784.1 [561.5;1194.2]	
Mean (std)	1205.4 (1833.0)	1472.0 (1517.2)		1076.4 (1537.6)	1303.3 (1470.5)	
**A.27**			0.0003			0.0197
Min / Max	5.0 / 5120.0	5.0 / 5120.0		5.0 / 5120.0	10.0 / 5120.0	
Med [IQR]	150.1 [46.5;817.6]	509.0 [309.1;910.9]		178.9 [109.5;851.8]	533.7 [204.2;1141.0]	
Mean (std)	603.1 (1003.4)	1047.6 (1406.3)		786.2 (1367.6)	1010.9 (1320.7)	
**Eta**			0.0001			0.0080
Min / Max	55.2 / 2098.2	94.6 / 5120.0		10.0 / 5120.0	5.0 / 2500.2	
Med [IQR]	196.5 [133.8;860.4]	612.4 [394.7;951.7]		268.2 [186.7;632.4]	552.7 [355.6;927.0]	
Mean (std)	488.3 (524.5)	848.2 (929.5)		629.5 (1012.6)	709.0 (480.5)	
**Delta**			0.0007			0.2459
Min / Max	10.0 / 5120.0	10.0 / 5120.0		5.0 / 2422.6	43.6 / 2083.5	
Med [IQR]	213.2 [133.4;694.3]	558.5 [333.9;900.6]		337.4 [234.8;814.7]	455.0 [332.4;755.8]	
Mean (std)	794.4 (1418.3)	804.4 (882.7)		553.3 (533.4)	572.5 (373.7)	
**Omicron BA.2**			0.0071			0.5432
Min / Max	10.0 / 5120.0	41.7 / 5120.0		5.0 / 1527.7	5.0 / 2006.5	
Med [IQR]	189.9 [148.1;551.8]	412.1 [225.5;572.9]		470.0 [235.7;648.6]	386.9 [269.1;556.6]	
Mean (std)	620.5 (1194.3)	526.3 (695.6)		517.6 (413.1)	432.4 (270.1)	
**XBB.1.5**			0.3233			0.2254
Min / Max	5.0 / 739.4	5.0 / 825.0		5.0 / 495.8	5.0 / 906.9	
Med [IQR]	80.5 [49.1;162.2]	101.6 [71.9;166.2]		167.5 [79.0;231.4]	140.6 [75.4;184.1]	
Mean (std)	129.8 (154.9)	123.9 (108.5)		173.3 (125.5)	154.2 (146.5)	

Table S11

Fold median over time for matched samples between matched HERITAGE aNN, HERITAGE aNP and LEGACY samples

	Comparison of match heritage aNN sample to LEGACY sample			Comparison of match heritage aNP sample to Legacy sample		
	**Heritage aNN sample**	**Legacy sample**	**Heritage aNN (ref: Lagacy)**	**Heritage aNP sample**	**Legacy sample**	**Heritage aNP (ref: Legacy)**
	**Fold median [95% CI]**	**Fold median [95% CI]**	**Ratio of fold median [95% CI]**	**Fold median [95% CI]**	**Fold median [95% CI]**	**Ratio of fold median [95% CI]**
**Wild type**						
Month 3 (ref: Baseline)	2.48 [1.62-3.83] ***	2.39 [1.89-2.84] ***	1.04 [0.64-1.67]	2.77 [1.98-4.00] ***	2.39 [1.88-2.83] ***	1.16 [0.77-1.74]
Month 6 (ref: Baseline)	2.10 [1.54-3.21] ***	1.62 [1.21-2.17] ***	1.30 [0.81-2.07]	2.25 [1.69-3.16] ***	1.62 [1.21-2.18] ***	1.39 [0.90-2.14]
Month 12 (ref: Baseline)	1.94 [1.31-5.59] ***	2.30 [1.73-3.33] ***	0.84 [0.38-1.87]	1.98 [1.43-2.88] ***	2.30 [1.72-3.33] ***	0.86 [0.53-1.39]
Month 6 (ref: Month 3)	0.85 [0.59-1.41]	0.68 [0.49-0.97] *	1.25 [0.72-2.17]	0.81 [0.62-1.04]	0.68 [0.49-0.98] *	1.19 [0.77-1.84]
Month 12 (ref: Month 3)	0.78 [0.51-2.33]	0.96 [0.70-1.48]	0.81 [0.35-1.90]	0.71 [0.53-0.97] *	0.96 [0.69-1.48]	0.74 [0.46-1.20]
Month 12 (ref: Month 6)	0.92 [0.58-2.50]	1.42 [0.95-2.27]	0.65 [0.28-1.51]	0.88 [0.69-1.12]	1.42 [0.94-2.29]	0.62 [0.37-1.03]
**A. 27**						
Month 3 (ref: Baseline)	23.18 [11.33-31.18] ***	5.26 [4.01-7.73] ***	4.41 [2.41-8.06] ***	3.91 [2.30-6.42] ***	5.26 [4.01-7.84] ***	0.74 [0.40-1.37]
Month 6 (ref: Baseline)	20.79 [9.81-33.83] ***	4.17 [3.21-7.69] ***	4.99 [2.34-10.64] ***	2.85 [1.72-4.73] ***	4.17 [3.20-7.76] ***	0.68 [0.35-1.34]
Month 12 (ref: Baseline)	30.44 [15.93-54.06] ***	7.85 [3.47-15.46] ***	3.88 [1.48-10.18] **	3.15 [1.76-5.39] ***	7.85 [3.52-15.41] ***	0.40 [0.16-1.01]
Month 6 (ref: Month 3)	0.90 [0.38-2.25]	0.79 [0.51-1.58]	1.14 [0.40-3.27]	0.73 [0.56-1.02]	0.79 [0.50-1.61]	0.92 [0.48-1.78]
Month 12 (ref: Month 3)	1.31 [0.66-3.21]	1.49 [0.59-3.38]	0.88 [0.27-2.85]	0.81 [0.53-1.22]	1.49 [0.62-3.40]	0.54 [0.21-1.40]
Month 12 (ref: Month 6)	1.46 [0.62-3.85]	1.88 [0.69-4.01]	0.78 [0.22-2.76]	1.11 [0.69-1.63]	1.88 [0.68-4.00]	0.59 [0.22-1.58]
**Eta**						
Month 3 (ref: Baseline)	7.27 [3.16-22.24] ***	2.50 [1.97-3.26] ***	2.91 [1.06-7.96] *	3.95 [2.40-5.27] ***	2.50 [1.98-3.26] ***	1.58 [0.99-2.52]
Month 6 (ref: Baseline)	7.10 [3.27-22.09] ***	2.31 [1.76-2.86] ***	3.07 [1.15-8.23] *	2.61 [1.68-3.32] ***	2.31 [1.76-2.85] ***	1.13 [0.74-1.72]
Month 12 (ref: Baseline)	10.01 [3.85-38.69] ***	2.71 [1.68-4.09] ***	3.69 [1.07-12.71] *	2.40 [1.56-3.29] ***	2.71 [1.68-4.17] ***	0.89 [0.49-1.60]
Month 6 (ref: Month 3)	0.98 [0.74-1.46]	0.92 [0.68-1.19]	1.07 [0.69-1.65]	0.66 [0.51-0.89] **	0.92 [0.67-1.18]	0.72 [0.48-1.07]
Month 12 (ref: Month 3)	1.38 [0.86-2.76]	1.08 [0.66-1.71]	1.28 [0.60-2.71]	0.61 [0.47-0.89] **	1.08 [0.66-1.71]	0.56 [0.32-1.00]
Month 12 (ref: Month 6)	1.41 [0.84-2.71]	1.17 [0.74-1.88]	1.21 [0.57-2.54]	0.92 [0.73-1.24]	1.17 [0.73-1.86]	0.79 [0.46-1.35]
**Delta**						
Month 3 (ref: Baseline)	17.72 [3.79-35.43] ***	16.51 [3.01-24.66] ***	1.07 [0.23-4.98]	3.32 [2.34-4.48] ***	16.51 [3.02-24.68] ***	0.20 [0.07-0.60] **
Month 6 (ref: Baseline)	17.83 [3.75-39.75] ***	16.18 [3.16-25.60] ***	1.10 [0.23-5.33]	2.65 [1.79-3.34] ***	16.18 [3.19-23.49] ***	0.16 [0.06-0.47] ***
Month 12 (ref: Baseline)	30.52 [6.21-63.81] ***	20.98 [3.94-35.47] ***	1.45 [0.29-7.21]	2.21 [1.59-2.93] ***	20.98 [3.94-35.67] ***	0.11 [0.03-0.33] ***
Month 6 (ref: Month 3)	1.01 [0.70-1.54]	0.98 [0.71-1.43]	1.03 [0.61-1.74]	0.80 [0.58-1.01]	0.98 [0.72-1.41]	0.82 [0.53-1.26]
Month 12 (ref: Month 3)	1.72 [1.01-2.96] *	1.27 [0.87-2.18]	1.35 [0.67-2.75]	0.67 [0.51-0.90] **	1.27 [0.87-2.19]	0.53 [0.31-0.91] *
Month 12 (ref: Month 6)	1.71 [0.87-2.86]	1.30 [0.90-2.08]	1.32 [0.63-2.73]	0.84 [0.68-1.11]	1.30 [0.90-2.09]	0.65 [0.40-1.05]
**Omicron BA 2**						
Month 3 (ref: Baseline)	2.61 [2.12-3.34] ***	12.42 [10.19-27.22] ***	0.21 [0.12-0.36] ***	2.15 [1.52-2.52] ***	12.42 [10.19-27.32] ***	0.17 [0.10-0.30] ***
Month 6 (ref: Baseline)	2.94 [2.57-4.83] ***	13.15 [10.16-30.74] ***	0.22 [0.12-0.42] ***	2.28 [1.67-2.67] ***	13.15 [9.84-30.74] ***	0.17 [0.09-0.32] ***
Month 12 (ref: Baseline)	7.86 [4.68-11.69] ***	15.70 [10.68-46.25] ***	0.50 [0.21-1.19]	2.10 [1.52-2.55] ***	15.70 [10.68-46.25] ***	0.13 [0.06-0.29] ***
Month 6 (ref: Month 3)	1.12 [0.99-1.73]	1.06 [0.76-1.49]	1.06 [0.68-1.64]	1.06 [0.87-1.38]	1.06 [0.76-1.50]	1.00 [0.66-1.51]
Month 12 (ref: Month 3)	3.01 [1.78-4.28] **	1.26 [0.82-2.25]	2.39 [1.23-4.65] *	0.98 [0.81-1.28]	1.26 [0.82-2.24]	0.78 [0.45-1.35]
Month 12 (ref: Month 6)	2.68 [1.42-3.59] *	1.19 [0.76-2.19]	2.25 [1.11-4.55] *	0.92 [0.76-1.19]	1.19 [0.75-2.18]	0.77 [0.43-1.38]

CI: confidence interval. P-value notation: p<0.05*; p<0.01**; p<0.001***

aNN: anti-N negative at baseline. aNP: anti-N positive at baseline

Supplementary materials and methods

HERITAGE study sites

Vaccine-seeking participants were recruited at four health facilities, including the Adabraka Polyclinic (Korle Klottey Municipal), the Greater Accra Regional Hospital (Osu-Klottey Sub-Metro of Accra Metropolitan Area), the Korle Bu Teaching Hospital (Ablekuma South Metropolitan District), and the University of Ghana Hospital, Legon (Ayawaso West Municipal District) (appendix p 2). All four health facilities are NHIS-accredited government/public hospitals with certification from the Ghana Health Service for the deployment of COVID-19 vaccines. A special subcohort, consisting of participants with clinically confirmed rheumatologic and autoimmune conditions, was recruited from the Rheumatology Unit of the Korle Bu Teaching Hospital.

The LEGACY clinical cohort

The Legacy study (NCT04750356) is a prospective observational cohort, established in January 2021 of adults undergoing PCR-based surveillance for SARS-CoV-2 across four institutions in northwest London. Extensive descriptions of the cohort can be found in our prior interim reports [21-24][1–4]. In the UK, from December 2020, healthcare workers, adults over 65 years, and those with either immunocompromise or caring responsibilities were offered a dose of COVID-19 vaccine. Participants were recruited to Legacy from January 2021, inclusion criteria were broad (adults employed by an institution using the Crick-COVID PCR testing pipeline and had undergone testing for SARS-CoV-2), exclusion criteria were limited to ability to provide written informed consent and willingness for longitudinal follow up and ongoing serological surveillance. Participants are followed up every six months. At each study visit, individuals gave details on any recent infection episodes, vaccination doses, and had blood drawn for serum for live-virus microneutralisation assays and anti-N IgG detection. Additional visits are conducted pre/post vaccination where possible and following a reported infection episode.

SARS-CoV-2 testing by RT-PCR for the HERITAGE study

the novel coronavirus (2019-nCoV) Nucleic Acid Diagnostic Kit (PCR-Fluorescence Probing) (Sansure Biotech Inc., Hunan Province, China) detects the *ORF1ab* gene and *N* gene of SARS-CoV-2 and the *RNase P* gene as an internal control. Oropharyngeal swab specimens were homogenised by vortexing, pulse spun, heat-inactivated at 65 °C for 10 min, and pulse spun to settle any debris. Subsequently, 10 µl of swab suspension was incubated with 10 µl of Sample Release Reagent (Sansure Biotech Inc., Hunan Province, China) for 10 min after gentle vortexing. For each reaction, 10 µl of swab suspension-release reagent mixture of extracted RNA (or negative control-release reagent mixture/positive control) was transported into a 0.2 ml PCR tube prefilled with 15 µl of master mix (13 µl of 2019-nCoV-PCR mix and 2 µl of 2019-nCoV-Enzyme mix). Negative and positive controls (provided in the kit) and blank controls (viral transport medium and nuclease-free water) were processed in each batch. The RT-PCR was run on a Molarray MA-6000 real-time quantitative PCR machine (Sansure Biotech Inc., Hunan Province, China). The thermocycler settings used for amplification include reverse transcription at 50 °C for 30 mins to produce cDNA; cDNA pre-denaturation at 95 °C for 1 min; denaturation at 95 °C for 15 sec; and 45 cycles of annealing, extension, and fluorescence detection at 60 °C for 30 sec. The thermocycler was left to cool at 25 °C for 10 sec. RT-PCR runs with a negative control with no Ct or Ct > 38 for all target channels and a positive control with characteristic sigmoidal amplification curves (S-shape) detected with Ct ≤ 35 for all three channels, were considered valid. Samples with sigmoidal curves for *RNase P* with a Ct value ≤ 38 were considered valid. Samples with sigmoidal curves for *ORF1ab* and/or *N* with Ct ≤ 38 were considered positive for SARS-CoV-2.

COVID-19 infection surveillance in the LEGACY study

The Crick-COVID PCR testing pipeline provided asymptomatic occupational health PCR based screening for employees of the Francis Crick Institute and a number of NHS hospitals, community trusts and care homes in the northwest London sector [25]. Legacy participants underwent weekly PCR surveillance (employer-mandated for Crick employees) from April 2020-Mary 2022. Subsequently infection surveillance in the Legacy cohort has been through participant reports of either a symptomatic episode or positive lateral-flow test. Following each report, same/next-day home based PCR confirmatory testing is conducted with subsequent sequencing to identify the infecting variant [26].

Analyses of live virus neutralisation titres

The median neutralisation level was compared across characteristics of the study participants using the Wilcoxon rank sum test for comparison between two groups and the Kruskal Wallis test for comparison between three or more groups. The Wilcoxon rank sum test is a non-parametric test used to compare the median of a non-normally distributed continuous variable between two different groups. The Kruskal Wallis test is also a non-parametric test and a higher level of the Wilcoxon rank sum test which is used to compare median of a non-normally distributed continuous variable between three or more groups [27].

Further analysis was performed to compare the neutralisation level at month 3, month 6, and month 12 to the neutralisation level at baseline using the fold median approach. Fold median was defined as the ratio of the median in one group to the ratio of the median in the comparative groups. The bootstrapping sampling approach was used to estimate the 95% confidence interval of the fold median.

For each of the six COVID-19 variants, the neutralisation was dichotomized to; neutralisation levels below 256, against neutralisation levels of 256 and above. The Pearson's chi-square test was used to assess the factors associated with neutralisation level above 256 over time. A binary logistic regression model, treating the type of vaccine as the major confounding variable, was used to assess the odds of neutralisation level above 256. Variables that were found significant from bivariate analysis were also considered as potential confounding variables in the final analysis. All statistical significance were considered at an alpha of 0.05 level.

To compare neutralisation data between the HERITAGE study and the LEGACY study samples were matched using Coarsened Exact Matching (CEM) to balance the two cohorts based on age, sex, vaccine type, the number of vaccine doses, and the time elapsed since the second dose [28]. Matching was performed with the R package 'MatchIt'. Each HERITAGE subject was matched to the most similar LEGACY subject and the rest was discarded (1-to-1 matching) [29]. For discrete variables like sex, vaccine type, and the number of doses, the samples were matched exactly. Age was grouped into 15-year intervals, and the time elapsed since the second dose was grouped into 35-day intervals. This process resulted in 357 matched pairs of samples.
The matching process was then repeated for the remaining unmatched samples, but with a less strict criterion, grouping the time elapsed since the second dose into 180-day intervals. This produced an additional 204 "fuzzy-matched" pairs of samples.

Data analysis and statistics

Data used in the LEGACY study were collected and managed using REDCap electronic data capture tools hosted at University College London [28]. Data were imported to R from REDCap prior to analysis and integrated using R Chronogram as previously described [29]. Data were manipulated, analysed and visualised using *tidyverse* R packages including *dplyr* and *ggplot2*. Summary descriptions of the cohort were generated using *gtsummary*. Continuous data were reported as the median value and interquartile range (IQR) or the first and third quartiles (Q1; Q3). Statistical tests were conducted with the *rstatix* R package.
The Quantum Geographic Information System (QGIS) version 3.28.2 was used to describe the location of the health facilities involved in this study.

Supplementary Materials Table 1: Summary of SARS-CoV-2 VOC/VOI used, their mutational profile and isolate source.

Lineage	Mutations present in Spike	Source
Ancestral [hCoV19/England/02/2020]	NA	Public Health England, UK
A.27	L18F, L452R, N501Y, A653V, H655Y, D796Y, G1219V	Dr. Jonas Fuchs and Prof. Dr. Hans-Georg Koch
Delta [MS066352H]	T19R, K77R, G142D, Δ156- 157/R158G, A222V, L452R, T478K, D614G, P681R, D950N	Prof. Wendy Barclay, Imperial College London, London, UK via the Genotype-to-Phenotype National Virology Consortium (G2P-UK)
Eta	Q52R, A67V, E484K, D614G, Q677H, F888L	Via G2P-UK
Omicron BA.2 [hCoV/England/FCI-179/2022]	T19I, Δ24-26, A27S, G142D, V213G, G339D, S371F, S373P, S375F, T376A, D405N, R408S, K417N, N440K, S477N, T478K, E484A, Q493R, Q498R, N501Y, Y505H, D614G, H655Y, N679K, P681H, N764K, D796Y, Q954H, and N969K	Francis Crick Institute
Omicron XBB.1.5	T19I, L24S, P25-, P26-, A27-, V83A, G142D, Y144-, H146Q, Q183E, V213E, G252V, G339H, R346T, L368I, S371F, S373P, S375F, T376A, D405N, R408S, K417N, N440K, V445P, G446S, N460K, S477N, T478K, E484A, F486P, F490S, Q498R, N501Y, Y505H, D614G, H655Y, N679K, P681H, N764K, D796Y, Q954H, N969K	Prof. Gavin Screaton, University of Oxford, Oxford, UK via the Genotype-to-Phenotype National Virology Consortium (G2P-UK)
